# Dual-Probe Activity-Based Protein Profiling Reveals Site-Specific Differences in Protein Binding of EGFR-Directed Drugs

**DOI:** 10.1021/acschembio.3c00637

**Published:** 2024-07-25

**Authors:** Wouter van Bergen, Kristina Žuna, Jan Fiala, Elena E. Pohl, Albert J.R. Heck, Marc P. Baggelaar

**Affiliations:** † Biomolecular Mass Spectrometry and Proteomics, Bijvoet Center for Biomolecular Research and Utrecht Institute for Pharmaceutical Sciences, University of Utrecht, Padualaan 8, Utrecht 3584 CH, The Netherlands; ‡ Netherlands Proteomics Center, Padualaan 8, Utrecht 3584 CH, The Netherlands; § Physiology and Biophysics, Department of Biological Sciences and Pathobiology, University of Veterinary Medicine, Wien, Vienna 1210, Austria

## Abstract

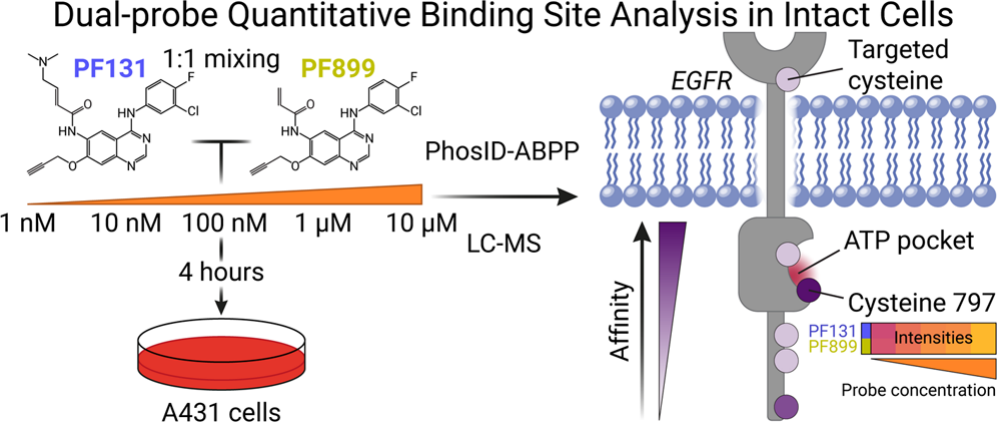

Comparative, dose-dependent analysis of interactions between small molecule drugs and their targets, as well as off-target interactions, in complex proteomes is crucial for selecting optimal drug candidates. The affinity of small molecules for targeted proteins is largely dictated by interactions between amino acid side chains and these drugs. Thus, studying drug–protein interactions at an amino acid resolution provides a comprehensive understanding of the drug selectivity and efficacy. In this study, we further refined the site-specific activity-based protein profiling strategy (ABPP), PhosID-ABPP, on a timsTOF HT mass spectrometer. This refinement enables dual dose-dependent competition of inhibitors within a single cellular proteome. Here, a comparative analysis of two activity-based probes (ABPs), developed to selectively target the epidermal growth factor receptor (EGFR), namely, PF-06672131 (PF131) and PF-6422899 (PF899), facilitated the simultaneous identification of ABP-specific binding sites at a proteome-wide scale within a cellular proteome. Dose-dependent probe-binding preferences for proteinaceous cysteines, even at low nanomolar ABP concentrations, could be revealed. Notably, in addition to the intrinsic affinity of the electrophilic probes for specific sites in targeted proteins, the observed labeling intensity is influenced by several other factors. These include the efficiency of cellular uptake, the stability of the probes, and their intracellular distribution. While both ABPs showed comparable labeling efficiency for EGFR, PF131 had a broader off-target reactivity profile. In contrast, PF899 exhibited a higher labeling efficiency for the ERBB2 receptor and bound to catalytic cysteines in several other enzymes, which is likely to disrupt their catalytic activity. Notably, PF131 effectively labeled ADP/ATP translocase proteins at a concentration of just 1 nm, and we found this affected ATP transport. Analysis of the effect of PF131 and its parent inhibitor Afatinib on murine translocase SLC25A4 (ANT1)-mediated ATP transport strongly indicated that PF131 (10 μM) partially blocked ATP transport. Afatinib was less efficient at inhibiting ATP transport by SLC25A4 than PF131, and the reduction of ATP transport by Afatinib was not significant. Follow-up analysis is required to evaluate the affinity of these inhibitors for ADP/ATP translocase SLC25A4 in more detail. Additionally, the analysis of different binding sites within the EGF receptor and the voltage-dependent anion channel 2 revealed secondary binding sites of both probes and provided insights into the binding poses of inhibitors on these proteins. Insights from the PhosID-ABPP analysis of these two ABPs serve as a valuable resource for understanding drug on- and off-target engagement in a dose- and site-specific manner.

## Introduction

Small molecule drugs interact with proteins, affecting protein conformation, activity, and protein–protein interactions.^1−4^ Drug–protein affinity is governed by interactions between the drug and amino acid side chains.^5^ Even subtle chemical modifications of drugs can markedly alter their affinity toward their protein targets, reshaping the target landscape.^6^ Moreover, many of the current drugs target pockets in protein structures, such as ATP- or GDP-binding pockets, that are to some extent conserved over different proteins in the proteome, leading to often undesired off-target binding.^7^ Therefore, the investigation of changes induced by subtle differences in the chemistry of the drugs on their targets is essential for drug development. Ideally, such investigations should be conducted at an amino acid resolution, allowing for a detailed exploration of the specific interactions between drugs and proteins.

Activity-based protein profiling (ABPP) coupled with peptide-centric enrichment methods enables the site-specific investigation of drug–protein interactions.^8−12^ ABPP utilizes activity-based probes (ABPs) to interrogate protein activity and/or (active) site occupancy.^13,14^ ABPs consist of a “warhead” to form a covalent bond with target proteins, a recognition element that improves affinity for specific proteins, and a reporter tag to enable visualization or enrichment of targeted proteins.^13,15−21^ A peptide-centric enrichment approach enables direct liquid chromatography–mass spectrometry (LC–MS) detection of ABP-bound peptides, resulting in a reduction of detected false positive identifications and a site-specific view of the ABP target landscape.^12^

The epidermal growth factor receptor (EGFR), a transmembrane receptor tyrosine kinase and a key player in the regulation of cell growth, is a validated target in cancer therapy.^22−25^ Dysregulation of EGFR signaling is linked to several diseases, including cancer, highlighting the importance of effective treatments that target this receptor, ideally with no off-target events.^6,22,26,27^ Recently, Lanning *et al.* introduced two selective Afatinib-derived EGFR-directed ABPs, PF-6422899 (PF899) and PF-06672131 (PF131; a dimethylaminomethyl (DMAM)-modified derivative of PF-6422899), enabling analysis of on- and off-target engagement by ABPP.^28^ The comparative protein-centric analysis of the target landscape of these two ABPs showed that DMAM substitution resulted in increased proteome-wide reactivity, which was partly attributed to the prolonged retention times in intact cells.^28^

Our study employed a peptide-centric ABPP approach using phosphonate-based enrichment tags (PhosID-ABPP) for the two ABPs in a single cellular proteome.^12^ Through their distinctive masses, we obtained a detailed view of the exact localization and relative labeling efficiencies of both ABPs over different concentrations, charting their site-specific binding interactions with proteins.

We were able to quantitatively monitor the binding sites of both probes simultaneously, even at a minimal probe concentration of 1 nM. This dual-probe binding site analysis uncovered diverse binding preferences for PF899 and PF131, even within single proteins. Our dose-dependent evaluation of binding sites allowed us to identify specific and nonspecific binding sites for both probes. The charted target landscape provides valuable insight into the effect of small modifications of EGFR inhibitors, enhancing our understanding of EGFR-directed protein–drug interactions.

## Results and Discussion

### Optimization of the Mass Spectrometry Settings on a TimsTOF HT for Targeted Analysis of ABP-Modified Peptides

**Figure 1:**
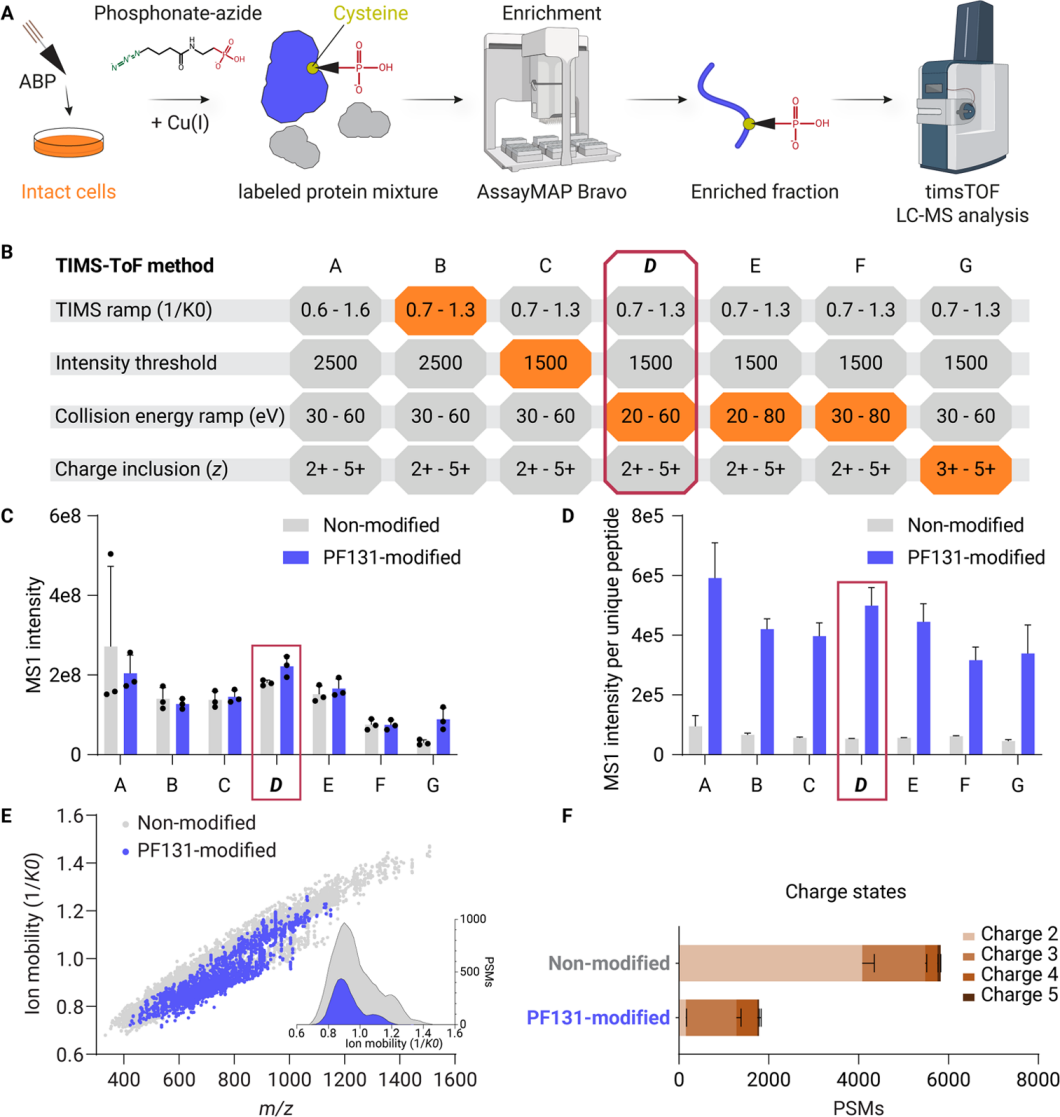
TimsTOF analysis enables efficient detection of activity-based PF131 binding sites. A) Overview of the site-specific proteomics workflow. B) A schematic of the tested variable parameters in the methods for the detection of PF131-modified (25 μM) peptides. All analyses were performed in triplicate (*n* = 3). C) Summed MS1 intensity of nonmodified and PF131-modified peptides using the different methods described in panel A. D) Summed MS1 intensity per unique peptide was calculated for both nonmodified and PF131-modified peptides. E) A plot displaying the ion mobility versus *m*/*z* of nonmodified peptides and PF131-modified peptides detected using method A. The inset displays the distribution of the ion mobility for nonmodified (gray) and PF131-modified (blue). F) Distribution of peptide-spectrum matches (PSMs) per charge state for both nonmodified and PF131-modified peptides.

This work builds further upon our recent report describing site-specific ABPP using phosphonate handles.^12^ To further improve the sensitivity of PhosID-ABPP and facilitate the comparative analysis of multiple ABPs within a complex proteome, we changed and optimized the settings on a timsTOF HT mass analyzer for enhanced identification and quantification of ABP-labeled peptides. Four distinct parameters were assessed for the detection of PF131 (25 μM) binding sites in intact A431 cells: 1) the separation and detection range of the trapped ion mobility spectrometry (TIMS) module, 2) the precursor intensity threshold for subsequent collision-induced dissociation (CID) and MS/MS analysis, 3) the collision energy range used for CID, and 4) the inclusion of charge states for MS/MS analysis (Figure 1B).

As a starting point, we used method A, which was optimized for the analysis of nonmodified tryptic peptides on the timsTOF HT platform (Figure 1A,B). Analysis of our pepsin-digested peptides using method A resulted in the detection of 1150 peptide-spectrum matches (PSMs) corresponding to 344 unique PF131-labeled peptides with a summed MS1 intensity of 2e^8^. In addition to these PF131-labeled peptides, 4433 PSMs derived from 2629 unique nonmodified peptides were concomitantly identified (summed intensity 2.7e^8^; Figures 1C and S1A,B), which, on average, indicates a significantly higher intensity for PF131-labeled peptides compared to nonmodified peptides (Figure 1D).

Next, we explored the use of ion mobility parameters to enhance the detection of ABP-labeled peptides by isolation/separation of ABP-labeled peptides, adopting a strategy that was previously successful in the analysis of post translational modifications (PTMs) and cross-linked peptides.^29,30^ Comparison of the ion mobility against the *m*/*z* for PSMs detected by method A revealed a clustering of PF131-modified peptides, spanning an ion mobility range of 0.7 to 1.3 1/*K*_0_. However, the cloud still displayed an overlap with the ion mobility space of nonmodified peptides (Figures 1E and S1C–G). Notably, the clustering of PF131-labeled peptides can be partially attributed to their higher charge states (Figure S1F). The elevated relative abundance of charge states 3+ or higher for PF131-labeled peptides, in comparison to their nonmodified counterparts, indicates an additional charge, potentially located on the nitrogen atom of the DMAM group of the ABP (Figures 1E and S1C–F). The observed range of the PF131-labeled ion mobility space prompted us to adjust the TIMS ramp from 0.7 to 1.3 1/*K*_0_ for the subsequently explored method (Figure 1B, methods B–G).

Shortening the TIMS range (method B) resulted in an overall decrease in signal for both PF131-modified and nonmodified peptides, possibly indicating a loss of some PF131-labeled peptides at the edges of the TIMS range. Nonetheless, lowering the MS1 precursor intensity threshold (method C) partially mitigated these losses. Further optimization of the ion mobility-dependent collision energy for CID using different ramps (methods C–F) indicated that method D (20 to 60 eV) was the most effective, resulting in the, on average, detection of 1489 PSMs with a summed intensity of 2.2e^8^ from 446 unique PF131-labeled peptides, along with the highest average hyperscore (Figures 1C and S1A,B,H).

Since PF131-modified peptides primarily exist in charge states 3+ or higher, we assessed method G, which exclusively selects precursors with charge states between 3+ and 5+ (Figure 1E,F). This approach reduced the detection of nonmodified peptides and retained most of the PF131-labeled peptides detected (Figures 1C,D,F and S1A–C). However, since the PF131-bound peptides derived from the main EGFR binding site could exist in charge state 2+ as well, we opted to utilize method D, in the remainder of this work, for the comparative site-specific detection of multiple ABPs in intact cells.

### Comparative Dose-Dependent Profiling of Two Distinct Activity-Based Probes in a Single Complex Proteome Reveals Probe-Specific Characteristics

**Figure 2:**
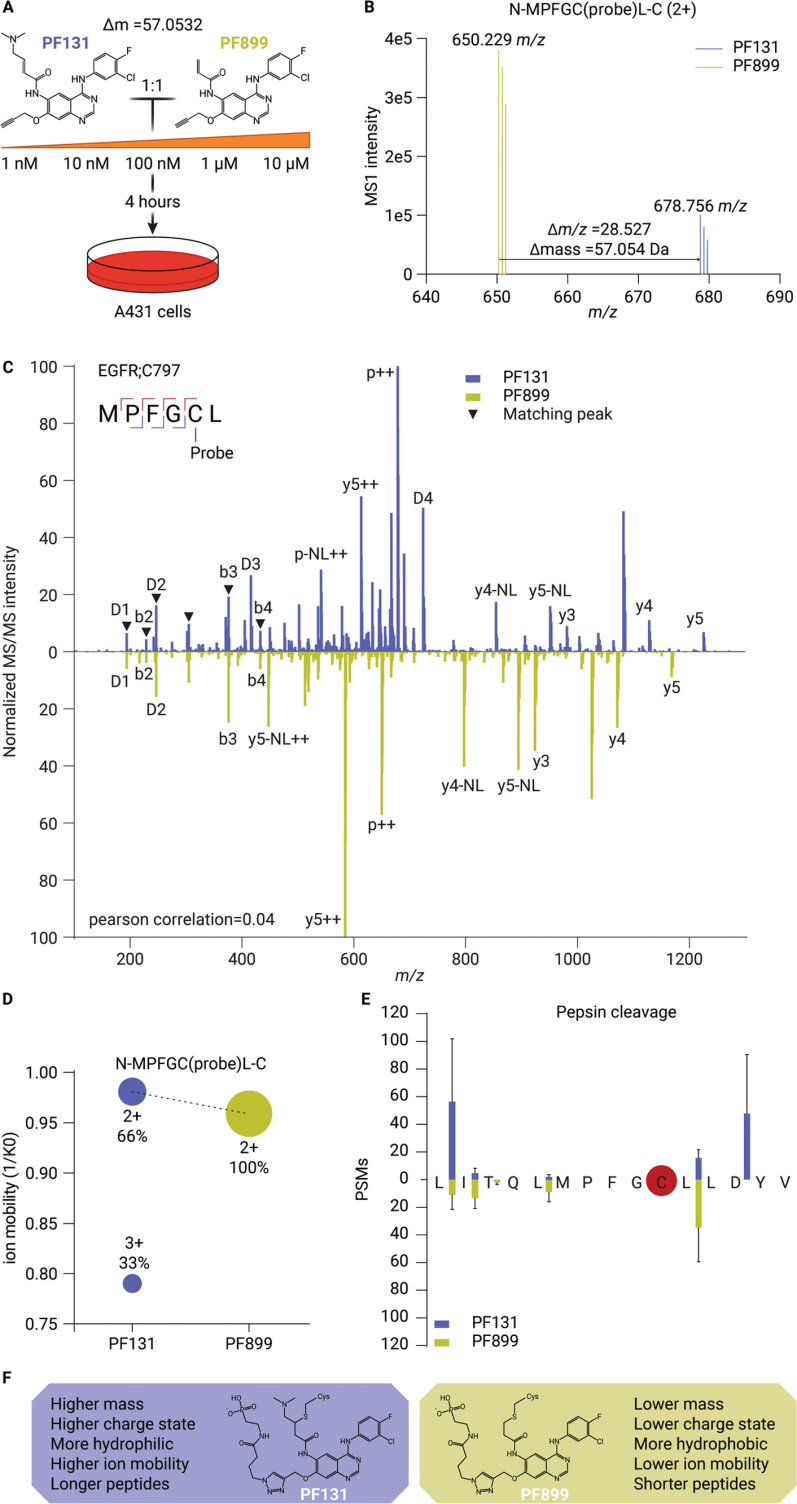
Comparative dose-dependent profiling of two activity-based probes in a single complex cellular proteome reveals probe-specific characteristics. A) Experimental design for the comparative dose-dependent profiling of two ABPs simultaneously in intact cells. A431 cells were treated with both PF131 and PF899 (mass difference of 57.0532 Da) mixed 1:1 ([PF131]: [PF899]) in growth medium at different concentrations for 4 h. PhosID-ABPP analysis was performed to identify the binding sites for both ABPs. Concentrations mentioned are the concentrations for individual probes and not the summed concentration. Experiments were performed in triplicate (*n* = 3). B) Combined MS1 signal of doubly charged MPFGC(PF131)L (blue) and MPFGC(PF899)L (yellow). The mass difference between the two peptides corresponds to the mass difference between the two ABPs. C) Mirror plot of MS/MS spectra of MPFGCL labeled with PF131 (blue) and PF899 (yellow), respectively. Matching and ABP diagnostic ions are marked with a black triangle. b, b-ion; y, y-ion; p, precursor; NL, neutral loss; D, diagnostic ion. D) Average ion mobility (1/*K*_0_) of MPFGCL bound to PF131 (blue) and PF899 (yellow) in different existing charge states. The size of the dot and the percentage indicate the proportional intensity of the charge state. E) Mirror bar graph displaying the peptide-spectrum matches (PSMs) corresponding to the pepsin cleavage site for peptides spanning the EGFR;C797 site bound to PF131 (blue) and PF899 (yellow). The data were obtained from the 10 μM ABP concentration. F) Summary of the general different characteristics observed between peptides bound to either one of the two ABPs in the liquid and gas phases.

Employing the optimized method for analyzing ABP-bound peptides, we concurrently investigated the dose-dependent site-specific interactions of two earlier introduced EGFR-directed ABPs, PF131 and PF899, in the context of a complete cellular A431 proteome (Figure 2A).

We selected the nonspecific protease pepsin for the proteolysis of ABP-labeled proteomes, based on previous findings demonstrating that pepsin, unlike trypsin, enables the detection of the primary ABP binding site of PF131 on EGFR.^12^ Here again, LC-MS analyses identified the covalent attachment of both ABPs to cysteine 797 (EGFR;C797), within the active site. This attachment was confirmed through the detection of multiple overlapping short peptide sequences encompassing this site, a general advantage when using less-specific pepsin instead of highly specific trypsin. To investigate and illustrate the specific characteristics of both ABPs in the liquid and gas phases, we evaluated the properties of a single peptide backbone (MPFGCL) that was bound by both probes. The mass difference of the peptide bound by the ABPs before fragmentation mirrored the mass difference between the two ABPs (Δ*m*/*z* = 28.527, Δmass = 57.054 Da, Figure 2B). Subsequent MS/MS analyses of the MPFGCL peptides bound to the ABPs exposed characteristic differences in fragment ions after CID. MS/MS fragment ions carrying the ABP adduct, or parts thereof, consistently showed *m*/*z* shifts that aligned with the mass difference between the two ABPs. In contrast, fragment ions without the ABP, and diagnostic ions derived from the phosphonate tag, matched perfectly between the MS/MS spectra of the differently labeled peptides (Figure 2C).

Analysis of the ion mobility profiles of the two ABPs revealed that PF131 exhibited a minor increase in 1/*K*_0_ for the 2+ charge state of the MPFGCL peptide, which is consistent with its larger molecular size (Figure 2A,D). We observed thatMPFGC(PF131)L was detected in both the 2+ and 3+ charge states, comprising 66% and 33%, respectively, of the total peak area of the peptide in MS1 scans (Figure 2D). In contrast, MPFGC(PF899)L was detected only as doubly charged ions (Figure 2D). This finding supports our hypothesis that PF131 has an extra positive charge located on its DMAM group, which is absent in PF899. The added hydrophilicity from the DMAM group manifested as a retention time difference between PF131 and PF899, with PF131-modified peptides eluting roughly 11 min prior to their PF899-modified counterparts (Figure S2C). The differences in retention time, ion mobility, and charge states between the ABP-modified peptides were consistent across all ABP-modified peptides and indicate that they are highly influenced by the properties of the ABP (Figures 2F and S2A–C).

Interestingly, our analysis elucidated differences in the pepsin cleavage patterns between peptides modified with the two ABPs. Inspection of all EGFR;C797-containing peptides revealed differences in the cleavage pattern at the C-terminus of the peptide sequence (Figure 2E). PF899-modified peptides were predominantly cleaved after leucine (L798), proximal to the ABP binding site, while the PF131-modified peptides indicated efficient cleavage after aspartic acid (D800) as well. Importantly, these differences in cleavage specificity affect or even prevent the relative quantification between two specific ABP-bound peptides. Due to these cleavage specificity variations, we suggest that the relative quantification of ABP binding efficacy between probes should be based on peptide populations that cover specific binding sites. Therefore, we calculated the summed MS1 intensity of all probe-labeled peptides for a specific site for relative quantification. This adjustment is expected to compensate for variations in the cleavage specificity. Moreover, given the observed differences in cleavage specificity, we advocate for the use of more and also nonspecific proteases in PhosID-ABPP to enhance quantification accuracy by preventing the signal intensity loss from specific ABP-bound peptides incompatible with proteases dependent on a singular specific cleavage site.

Taken together, the timsTOF HT enabled effective detection of ABP binding events from dual probe-labeled intact cells, revealing significant differences in the liquid and gas phase properties of ABP-labeled peptides, as summarized in Figure 2F, which facilitated robust and reproducible detection and quantification of their respective binding sites, further motivating an in-depth exploration of these binding sites at lower concentrations.

### Dose-Dependent Site-Specific Target Landscapes Reveal Probe-Specific Target Engagement in Intact Cells

**Figure 3:**
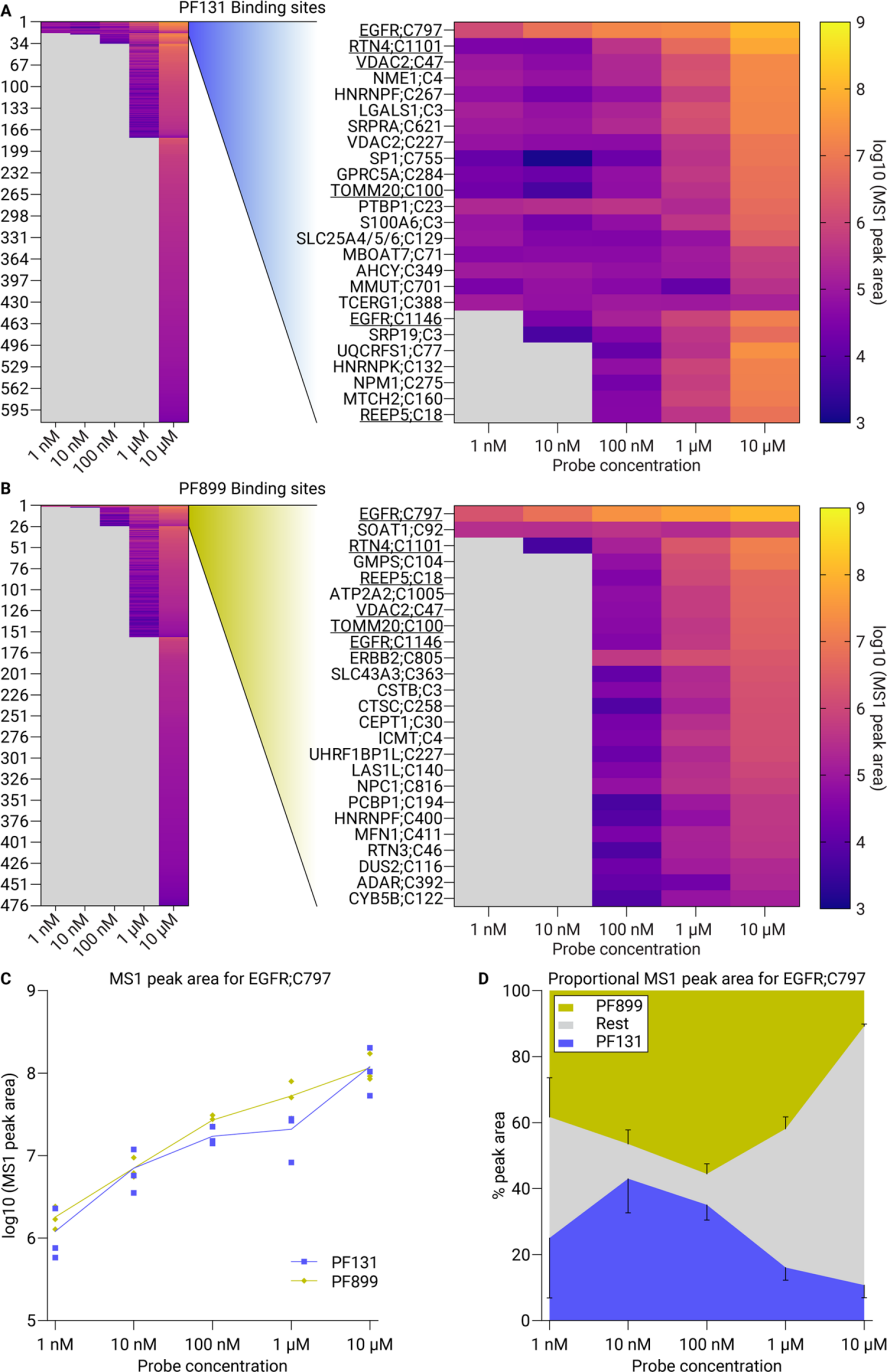
Dose-dependent site-specific target landscape reveals probe-specific target engagement in intact cells. A,B) Heatmaps displaying the average aggregated MS1 peak areas of 613 and 476 PF131 and PF899 binding sites at different concentrations in A431 cells, respectively (left segment). The right segments show a zoom of the top-25 binding sites. Underlined sites are the top-25 targets for both PF131 and PF899. C) Line plot of the aggregated MS1 peak areas for binding of PF131 (blue) and PF899 (yellow) to EGFR;C797 across the concentration range. D) Density plot of the proportional aggregated MS1 peak areas (*i.e.*, the intensity of the specific site as a percentage of the total probe-labeled intensity in the LC–MS experiment) for the binding of PF131 (blue) and PF899 (yellow) to EGFR;C797 and other sites (gray) across the concentration range.

Analysis of the concentration-dependent competitive ABP labeling experiment revealed excellent sensitivity of the site-specific ABPP strategy with PSMs, including those corresponding to PF131-bound EGFR;C797, being detected at a probe concentration as low as 1 nM (Supporting Information Data 1 and 2). To quantify probe-specific concentration-dependent binding events, we generated a library of identified ABP-bound peptides at a 10 μM probe concentration for both PF131 and PF899. Data analysis in Skyline-daily enabled extrapolation of the corresponding MS1 signals at lower concentrations and quantification of ABP-bound peptides across the full concentration range.^31^ The MS1 peak areas for all ABP-bound peptides corresponding to specific sites were aggregated and allowed quantification of 613 PF131- and 476 PF899-binding sites at a probe concentration of 10 μM (Figure 3A,B). Substantial off-target binding at a 10 μM ABP concentration was found for both probes. In line with prior research, we observed that PF131 is more reactive than PF899.^28,32^ Notably, probe labeling efficiency not only reflects the intrinsic binding affinity of the probe for specific protein sites but is also influenced by factors such as probe stability, cellular uptake of the probe, and intracellular probe distribution. At lower ABP concentrations, the target landscapes for both probes were more restricted. Interestingly, at low ABP concentrations, PF899 appeared to be much more selective, with only two targets (EGFR and SOAT1) detected, while 18 distinct target proteins were detected for PF131 at a 1 nM probe concentration. (Figure 3A,B).

Exploration of the hyperreactivity of the top 25 sites for both PF131 and PF899 in A431 cells, referenced to the CysDB database, indicated only 4 hyperreactive cysteines among these sites (Supporting Information Data 2). This suggests that the ABP affinity is not solely directed by cysteine reactivity.^35^

The concentration-dependent analysis was also conducted in low-EGFR-expressing A549 cells. In this alternative cell line, PF131-binding to EGFR;C797 was also detected and quantified over a broad concentration range. Moreover, the increased promiscuity of PF131 compared to PF899 was recapitulated in this cell line, with 563 and 87 quantified binding sites at a 10 μM probe concentration, respectively (Figure S3A,B). The origin of the relatively low number of PF899 binding sites (87) in A549 cells compared to A431 cells (431) remains to be investigated. The smaller number of detected binding sites at lower ABP concentrations also applies to A549 cells, with some overlap in the observed binding sites, for instance, PF131-bound VDAC2;C47 and PF899-bound GMPS;C104 at probe concentrations of 10 and 1000 nM, respectively (Figure S3C,D). 265 PF131 sites were identified in both A431 and A549 cells, indicating that the ABPP findings are to a considerable extent transferable to other cell lines.

As anticipated, EGFR;C797 consistently displayed the highest intensity of all binding sites throughout the concentration range for both ABPs in A431 cells, indicating a high specificity of the probes for their intended primary target (Figure 3A,B). Comparing the concentration-dependent binding of PF131 and PF899 to EGFR;C797 indicated no clear preference for either probe, as both displayed a similar intensity increase across the probe concentration range (Figure 3C). Of note, the proportional intensity of EGFR;C797 (*i.e.*, the MS1 peak area of the specified site as a percentage of the total probe-labeled MS1 peak area in the LC–MS experiment) reached its maximum for both probes at 10 and 100 nM (Figures 3D and S4A). Off-target labeling increased substantially with concentrations of the probes exceeding 100 nM, mainly caused by PF131’s higher reactivity, suggesting that EGFR-directed inhibitors lacking the DMAM group are superior in selectively inhibiting the EGFR (Figures 3D and S4A,B). These results indicate a specificity range of 1 to 100 nM for these probes and suggest binding saturation of EGFR’s ATP pocket above 100 nM in A431 cells, consistent with the approximate IC_50_ of these ABPs (namely, 84 nM for the nonalkynylated derivative of PF899) which was also performed in A431 cells.^28^

In conclusion, the concentration-dependent and ABP-specific analysis of binding site engagement resulted in the detection of over 450 quantified binding sites per ABP. Although both probes show widespread nonspecific reactivity in the micromolar range, with PF131 being more promiscuous, they exhibit good specificity for EGFR’s ATP pocket in the nanomolar range. Nevertheless, both probes engage in interactions with other binding sites in the nanomolar range, which require further investigation.

### Concentration–Response Analysis of ABP Binding Sites Elucidates ABP Binding Preferences in the Low Nanomolar Range

In-depth, concentration-dependent analysis revealed differences in target landscapes between PF131 and PF899 in the nanomolar range. The analysis identified multiple sites with differential binding detection onsets as low as 1 nM, with several sites being unique for individual ABPs, indicating distinct binding preferences.

**Figure 4:**
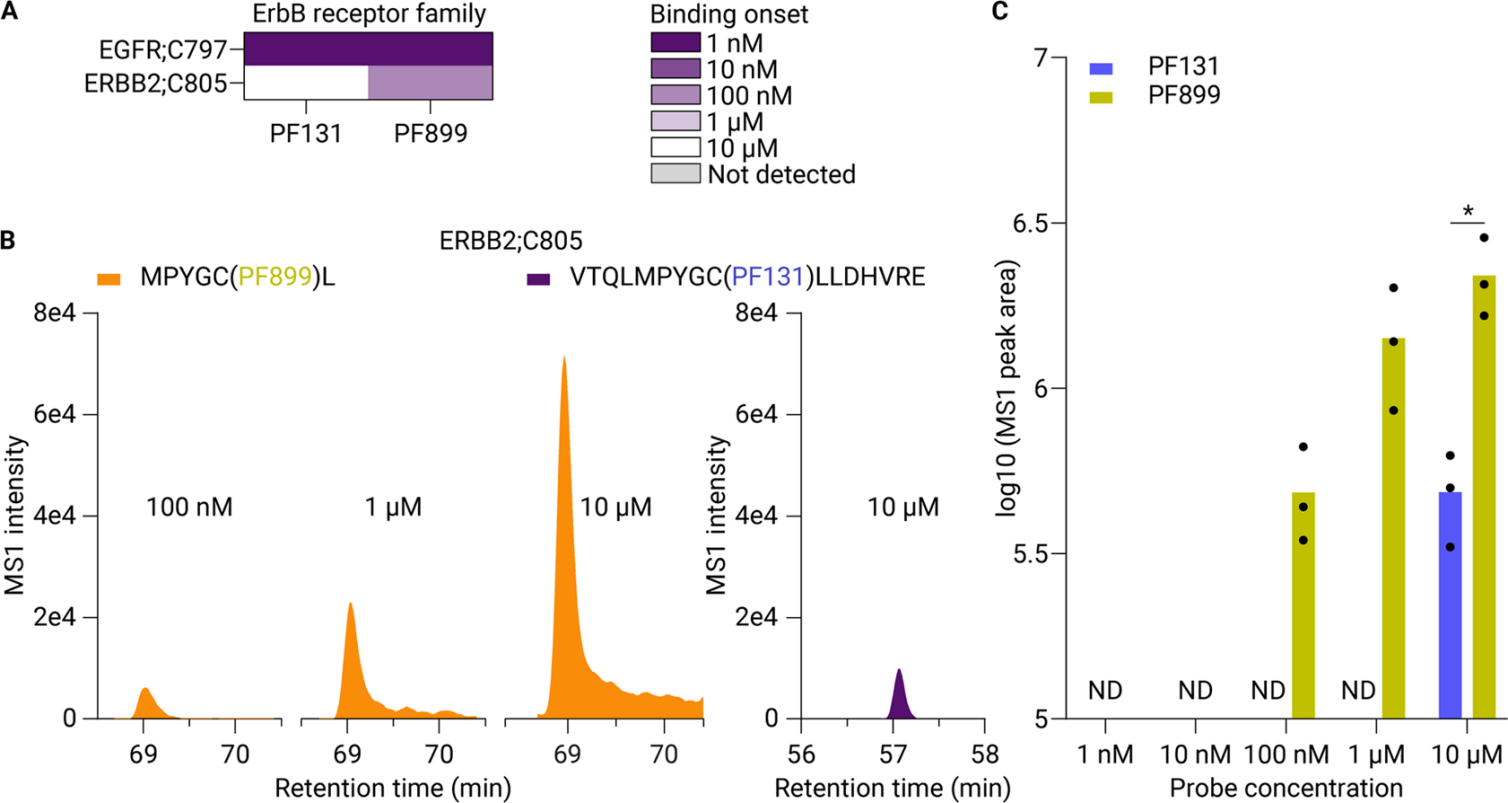
Dose-dependent profiling of ABP binding sites reveals that the ERBB2’s active site is relatively favored by PF899. A) Binding onset table displaying the ABP concentration at which the ABP initiated binding toward active site cysteines in EGFR and ERBB2. Experiments were performed in triplicate (*n* = 3). B) MS1 peak areas of MPYGC(PF899)L (orange) and VTQLMPYGC(PF131)LLDHVRE (purple) corresponding to both ABPs binding toward ERBB2;C805. C) Bar graph of the aggregated MS1 peak areas for PF131 (blue) and PF899 (yellow) binding to ERBB2;C805 across the concentration range. * indicates a significant difference in a paired *t* test at the *p* = 0.05 level. ND, not detected.

Our data revealed that ERBB2;C805 is targeted by both PF131 and PF899, with binding commencing at 10 μM and 100 nM, respectively (Figure 4A). ERBB2 is a member of the ErbB receptor tyrosine kinase family, to which EGFR also belongs. The binding pocket of its active site cysteine (C805) is homologous to that of EGFR (C797).^33^ Interestingly, while the signal intensity of labeled EGFR;C797 was similar for both probes across all concentrations, the MS1 peak areas of peptides encompassing ERBB2;C805 revealed that the peak area of PF899 bound to ERBB2;C805 was larger than that of PF131 bound to ERBB2;C805 at a probe concentration of 10 μM (Figure 4B). Furthermore, MS1 peak areas for PF899-bound ERBB2;C805 showed a dose-dependent increase from 100 nM to 10 μM. These results could be extrapolated to the sum of the total peptide population covering ERBB2;C805 for both ABPs, showing an earlier binding onset and significantly higher binding intensity at a 10 μM probe concentration of PF899 to ERBB2;C805 compared to PF131 (Figure 4C). Prior research did not indicate a preference for PF899 binding to ERBB2, as both PF131 and PF899 bound with similar intensity at 1 μM.^28^ We suspect that this difference may be attributed to variations in the study designs. While direct competition between two ABPs at different concentrations in intact cells can reveal binding preferences, separate labeling experiments at a single ABP concentration might not show these competition-induced preferences. The tendency of PF899 to favor ERBB2’s ATP pocket might arise from a decreased affinity when the DMAM moiety is introduced to PF131. This effect might also be seen with other EGFR-directed inhibitors.

**Figure 5:**
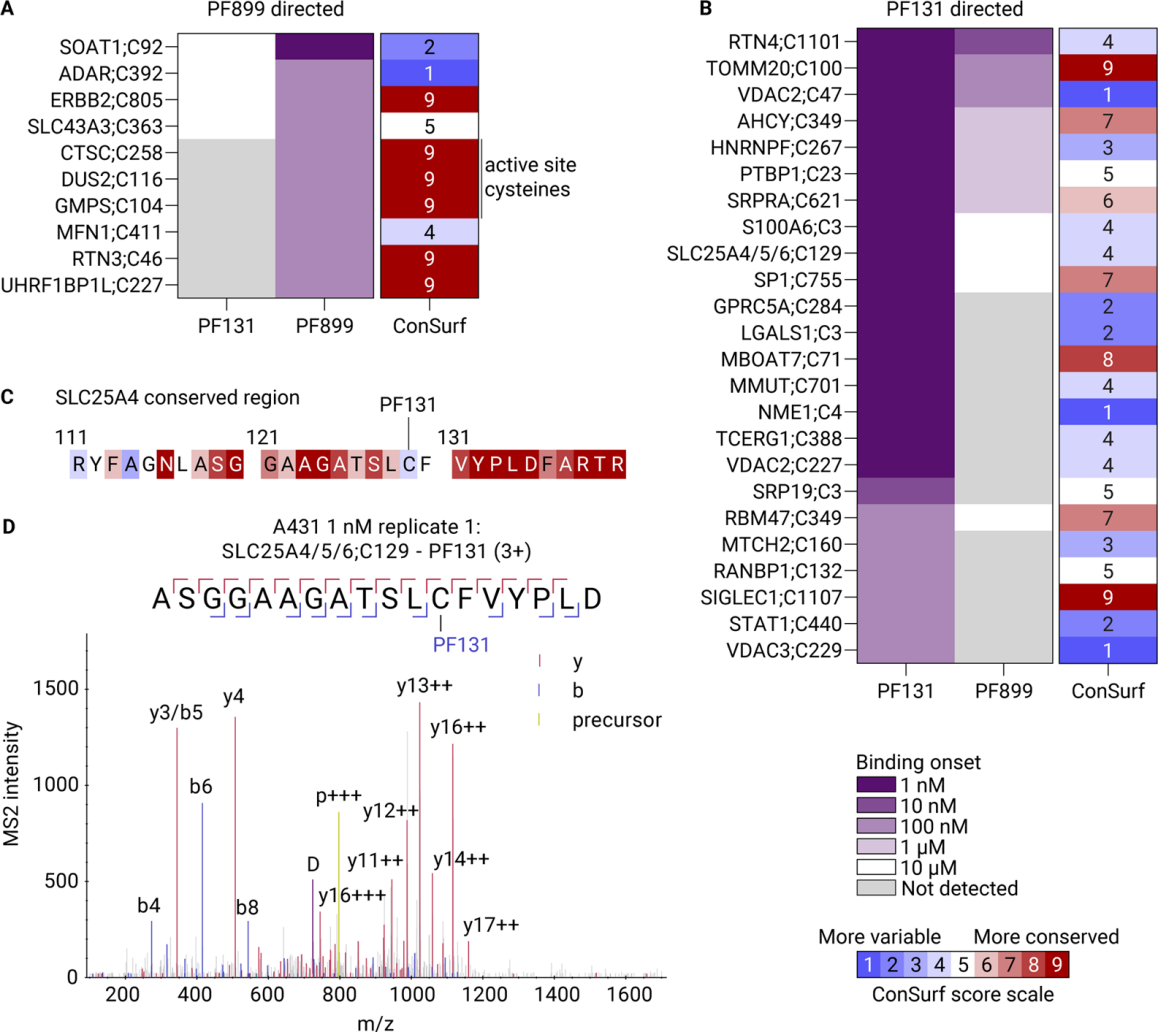
Analysis of off-target ABP binding sites in the nanomolar range elucidates binding preferences for both probes. A) Binding onset tables displaying the ABP concentration at which the ABP initiated binding toward the cysteine for the PF899-directed (A) and PF131-directed (B) sites. The conservation of the targeted cysteine is expressed in the third column as the ConSurf score. C) ConSurf analysis of the region surrounding PF131-targeted SLC25A4;C129. D) Experimental MS/MS evidence for binding of PF131 to cysteine 129 on SLC25A4/5/6. b: b-ion, y: y-ion, p: precursor, and D: diagnostic ion.

PF899 binds, in addition to EGFR;C797, to SOAT1;C92 at a 1 nM probe concentration and does not show an increase in labeling intensity with increasing concentrations, suggesting binding saturation at 1 nM. SOAT1’s (sterol O-acyltransferase 1) cysteine 92 is ligandable by an electrophilic scout fragment, KB05; however, the implication of this binding remains elusive.^34,35^ ConSurf scores of binding sites show that PF899 targets six conserved cysteines at nanomolar concentrations (the ConSurf score scale ranges from 1 to 9, with a higher score meaning a more evolutionarily conserved amino acid) (Figure 5A).^36^ While RTN3;C46 and UHRF1BP1L;C227 are predicted to be structural cysteines and are not known to perform additional functions, three cysteines that are exclusively targeted by PF899 are known as active site cysteines (CTSC;C258, DUS2;C116, and GMPS;C104). Consistent with our data, tRNA-dihydrouridine synthase (DUS2) was previously identified as a preferred PF899 target over PF131, whereas cathepsin C (CTSC) and GMP synthase (GMPS) were not described as PF899-preferred proteins.^28^

Cathepsin C is a lysosomal exocysteine protease, with a critical cysteine in its catalytic dyad, which is targeted by PF899 at a 100 nM probe concentration. This protease plays a key role in inflammatory pathways, activating serine proteases in neutrophil granules through its cleavage function.^37^ Cathepsin C is a therapeutic target since its overactivity may lead to disorders caused by hyperreactive neutrophils, such as noncystic fibrosis bronchiectasis or COVID-19-induced inflammatory diseases.^37^ X-ray crystallography of inhibitor-bound CTSC demonstrated covalent binding to cysteine 258, implying that binding at this site renders the protease inactive.^38^ Moreover, lysosomal accumulation of small molecule EGFR inhibitors has been proposed to cause increased engagement with cathepsins.^6,39^ PF899’s binding to this site and PF131’s lack thereof may suggest that PF899 accumulates more in the lysosome than PF131, and affects cathepsin C activity.

GMP synthase, another PF899 target, is a potential target for anticancer and immunosuppressive therapies.^40−42^ GMP synthase catalyzes the amination of xanthosine 5′-monophosphate to produce GMP.^42^ During catalysis, glutamine is hydrolyzed by cysteine 104, the PF899 binding site, to generate the amino group needed for the amination reaction. The glutamine hydrolysis can be uncoupled from GMP synthesis, as other nitrogen sources can also be used. PF899’s binding toward GMPS;C104 shows that PF899 probably inhibits glutamine hydrolysis of GMPS, without interfering with the GMP synthetase function of the enzyme, similar to Acivicin.^42^

PF131 targets a broader range of proteins at nanomolar concentrations than does PF899 (Figure 5B). Several conserved cysteines with ConSurf scores >7 are targeted by PF131 at these concentrations. All of these cysteines are predicted by ConSurf to be buried and perform a structural function in the protein; for example, cysteine 1107 in SIGLEC1 was predicted to form a disulfide bridge with cysteine 1149.^43^

We previously reported PF131’s affinity for Reticulon-4 at cysteine 1101 in intact cells with a concentration of 25 μM.^12^ Our current results reveal that RTN4 binding by PF131 can even be detected at a concentration as low as 1 nM and 10 nM by PF899. MS1 peak areas show that RTN4;C1101 is a preferred binding site for PF131, consistently showing higher intensities than PF899 across the concentration range (Figure S5A).

Intriguingly, we detected multiple PSMs in all replicates that provided evidence for PF131’s binding toward cysteine 129 on SLC25A4/5/6 at the 1 nM ABP concentration (Figure 5D), and we identified this site in both A431 and A549 cells. These proteins are mitochondrial ATP/ADP transporters, facilitating ADP’s movement into the mitochondria and ATP’s export. While PF131 exhibited binding at the lowest concentration, PF899 only binds at 10 μM, suggesting a superior affinity of PF131 for these proteins. Structural insights reveal that cysteine 129 is positioned within an α-helix spanning the mitochondrial membrane, which, in combination with other helices, forms a pore for ATP in the M-state and ADP in the C-state.^44^ Though cysteine 129 is not a conserved residue, with a ConSurf score of 4, the surrounding region is highly conserved and involved in binding of ATP or ADP (Figure 5C). Both M- and C-states can be inhibited by bongkrekic acid and carboxyatractyloside, respectively, and can cause severe toxic effects at low concentrations.^45,46^ PF131’s binding to mitochondrial ATP/ADP transporters might disrupt their function, leading to adverse events. The DMAM moiety in EGFR-directed inhibitors appears to provide improved affinity toward these antiporter proteins, and this valuable information could be used when designing novel drugs for ATP/ADP antiporters.

### PF131 and Afatinib Block ATP Transport by SLC25A4 (ANT1)

**Figure 6:**
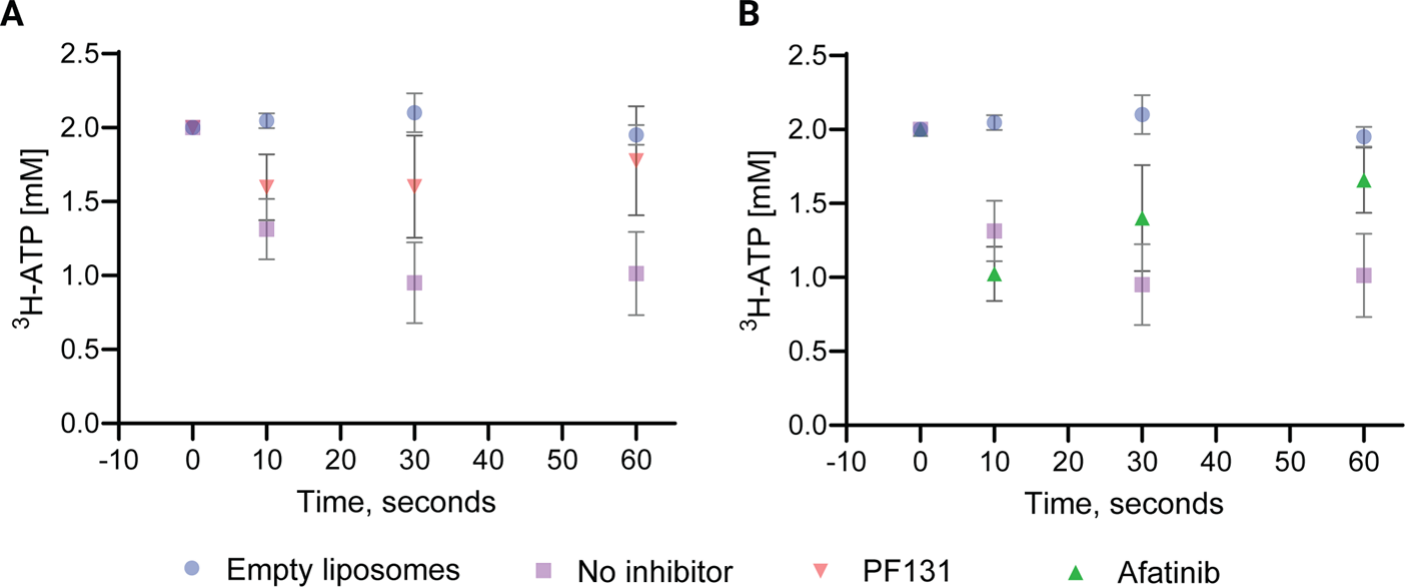
Decrease of ^3^H-ATP concentration upon initiation of the ATP/ADP exchange in proteoliposomes containing reconstituted SLC25A4 (squares). A) ATP transport was not present in empty liposomes (circles), active transport was observed in proteoliposomes containing reconstituted SLC25A4 (ANT1), and transport was inhibited by 10 μM PF131 (red triangles). B) Transport was also partly inhibited by 10 μM Afatinib (green triangles). Data are the mean ± SD of at least three independent experiments.

To corroborate our findings and investigate whether PF131 can indeed block transport of ATP by SLC25A4, we conducted an ADP/ATP transport assay using purified murine SLC25A4 reconstituted in liposomes. SLC25A4 was expressed in *E. coli* and purified according to an established protocol (Figure S8).^47^ ANT1-containing proteoliposomes filled with 2 mM ATP and 2 mM ^3^H-labeled ATP were prepared, and ATP transport was monitored by scintillation counting of ATP remaining in liposomes after 0, 10, 30, and 60 s (Figures 6 and S7).

We first investigated whether we could measure SLC25A4-mediated transport of ADP/ATP from the liposomes. For this purpose, we compared ADP/ATP transport among SLC25A4-loaded liposomes, empty liposomes, and liposomes after the addition of known blockers of SLC25A4-mediated ADP/ATP transport, combined carboxyatractyloside (CATR) and bongkrekic acid (BKA), each at 10 nM (Figure S7). Active ADP/ATP transport was observed for SLC25A4-loaded liposomes, and the calculated transport rate was 0.72 ± 0.28 μMol of 3H-ATP/(s*(mg protein)), whereas it was completely absent (0) in empty liposomes or liposomes treated with BKA and CATR, confirming our ability to measure SLC25A4-mediated ADP/ATP transport (Figure S8).

We then investigated whether PF131 and its parent inhibitor, Afatinib, could inhibit SLC25A4-mediated ADP/ATP transport (Figure 6A,B). We observed that PF131 was more effective at reducing ATP transport compared to Afatinib. PF131 reduced ATP transport at all time points (*t* = 10, 30, and 60 s). Although no significant difference in ATP transport between active and PF131-inhibited transport was measured for individual time points (Figure 6A), a *t* test analysis of the ^3^H-ATP remaining in the liposomes over all time points revealed a significant difference between the active transport (mean ± SD [^3^H-ATP, mM] = 1.66 ± 0.1) and PF131 inhibition (mean ± SD [^3^H-ATP, mM] = 1.09 ± 0.19). The difference of 0.57 ± 0.123 mM (*p* = 0.01) strongly indicates (partial) inhibition of ATP transport by PF131.

Inhibition by Afatinib is less pronounced, and we observed no reduction in ATP transport after 10 s, but the ATP transport at 30 and 60 s appears to be reduced by Afatinib inhibition compared to control-treated liposomes. However, a comparison of the means over all time points does not reveal a significant difference between the controls and Afatinib-inhibited ATP transport. Follow-up studies are required to explore the extent to which Afatinib inhibits SLC25A4-mediated ATP transport and to determine the binding affinities of both inhibitors toward the ADP/ATP translocase SLC25A4.

Taken together, these results strongly indicate that PF131 reduces the level of SLC25A4-mediated ATP transport. Furthermore, the site-specific PhosID-ABPP can identify new targets on which the probes have a functional effect. Additionally, our site-specific assay can help in predicting whether specific interactions between the probe and targeted proteins may affect protein function.

### Analysis of Multiple Probe Binding Sites within Individual Proteins

**Figure 7:**
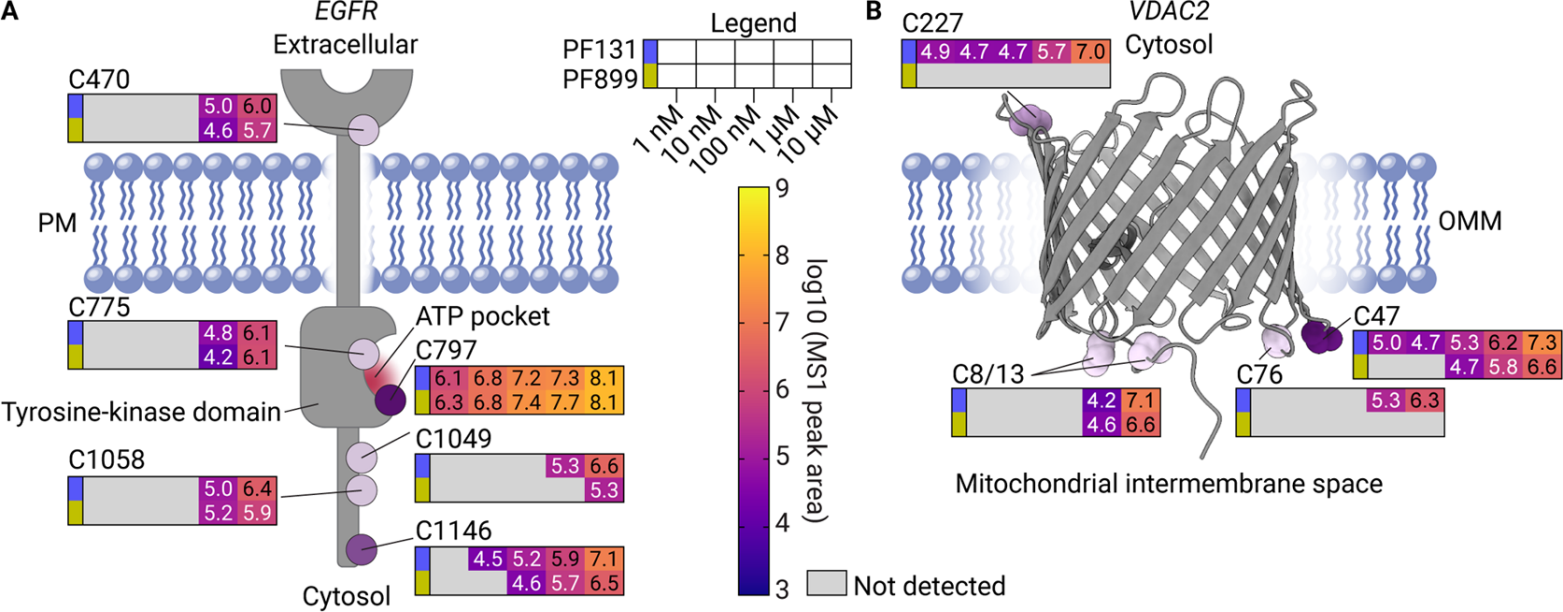
Binding preferences of the two probes within individual proteins. A) Topology of EGFR displaying the average aggregated log10 MS1 peak areas at different ABP concentrations on each binding site for PF131 (blue) and PF899 (yellow). PM: plasma membrane. B) Structural model for porcine VDAC2 displaying the average aggregated log10 MS1 peak areas at different ABP concentrations on each binding site for PF131 (blue) and PF899 (yellow), adapted from Leung et al., 2021, PDB: 7NIE.^50^ OMM, outer mitochondrial membrane.

Site-specific analysis facilitates the discovery of multiple ABP binding sites within individual proteins. Within EGFR, five distinct ABP binding sites were identified in addition to the anticipated target C797 in the ATP binding pocket. Four of these sites are localized in the cytosolic domain (Figure 7A). The primary binding site (C797) and cysteine 775, which are both solvent-accessible cysteines localized in the ATP pocket, display substantial disparities in their measured labeling intensities.^48^ ABP-modified cysteine 797 can be detected at concentrations as low as 1 nM by both probes and consistently displays higher labeling intensities compared with C775 throughout the whole concentration range. This observation is in line with the binding pose of covalent chlorofluoroacetamide-based EGFR inhibitors, with the Michael acceptor situated near cysteine 797.^33,48^ Except for cysteine 1146, binding to EGFR cysteines other than C797 is detectable only at ABP concentrations of 1 μM and above. C1146-binding by PF131 and PF899 was already detected at 10 and 100 nM, respectively. This pattern suggests differential binding affinities across different EGFR cysteines, with both ABPs demonstrating relatively efficient binding to cysteine 1146 compared to the other nonprimary binding sites in EGFR. Relatively efficient binding to cysteine 1146 may be potentially explained by increased solvent accessibility and/or the interactions that the ABP establishes with the protein near this site.

Additionally, voltage-dependent anion channels 1, 2, and 3 were found to exhibit ABP binding sites, with voltage-dependent anion channel 2 (VDAC2) exhibiting five distinct probe binding sites (Figures 7B and S6), which are identified in both A431 and A549 cells, demonstrating that our site identification method is consistent in different cell lines. In line with previous studies on cysteines in VDAC isoforms, totally reduced and redox-active cysteines in VDAC1, 2, and 3 are primarily targeted by both probes.^49^ Mapping the bound cysteines on an electron microscopy model of porcine VDAC2 shows that most of these cysteines are located in the mitochondrial intermembrane space (Figure 7B).^50^ Notably, cysteine 47 displayed the highest labeling intensity by both ABPs, with PF131 and PF899 binding being detected at 1 and 100 nM ABP concentrations, respectively (Figures 7B and S5B). This suggests an ABP binding pose with the Michael acceptor of the ABPs proximal to C47. Furthermore, CysDB was used to assess the reactivity of cysteines in VDAC2. This analysis revealed that VDAC2 has two known hyperreactive cysteines, namely C210 and C227.^34,35,51^ Only C227 bound to PF131 was detected in our experiments, which points toward labeling of the cysteines driven by ABP affinity for specific VDAC2 sites rather than just cysteine reactivity.

In summary, these findings demonstrate the binding of both probes at different cysteines within individual proteins, revealing secondary binding sites and providing insights into the preferred binding orientations of ABPs within proteins. Importantly, differentiating between primary and secondary binding sites of small molecule drugs in proteins is unattainable without site-specific ABPP. Furthermore, binding to secondary sites within proteins can elicit functional consequences, making it an essential aspect to consider during drug binding evaluation by ABPP.

## Conclusions

In this study, we extended upon our earlier research on site-specific activity-based protein profiling using phosphonate handles, integrating simultaneous dose-dependent competition of two activity-based probes in intact cells. To identify optimal settings for the detection of ABP-bound peptides on the timsTOF HT, we leveraged the unique characteristics of ABP-bound peptides compared to unmodified counterparts to enhance their detection. Furthermore, we discovered specific properties for each probe across multiple dimensions within a single LC–MS/MS run including reversed-phase liquid chromatography, ion mobility separation, enzymatic proteolysis, and collision-induced dissociation. The detailed deconvolution of ABP binding sites allowed for the relative quantification of the binding efficiency to cysteine residues across the total proteome for two EGFR-directed probes. Notably, the probe labeling efficiency of these covalent ABPs not only reflects the intrinsic binding affinity of the probe for specific protein sites but also is influenced by factors such as probe stability, cellular uptake of the probe, and intracellular probe distribution. While both probes showed similar labeling efficiency for the EGF receptor, PF131 displayed a broader off-target profile at all concentrations. PF899 displayed a higher labeling intensity for the ERBB2 receptor and bound specifically to catalytic cysteines in CTSC, DUS2, and GMPS, likely disrupting their enzymatic activity. Our analysis revealed that PF131 labels mitochondrial ADP/ATP translocases from a concentration of just 1 nM by binding to cysteine 129 of SLC25A4/5/6. We investigated whether the interaction of PF131 and its parent inhibitor Afatinib with ADP/ATP translocase SLC25A4 affected ATP transport. Our analysis of the effect of PF131 and Afatinib on murine ADP/ATP translocase SLC25A4 (ANT1)-mediated ATP transport strongly indicated that PF131 (10 μM) partially blocked ATP transport. Afatinib was less efficient at inhibiting ATP transport by SLC25A4 compared to PF131, and the reduction of ATP transport by Afatinib was not significant. Follow-up analysis is required to evaluate the affinity of these inhibitors for the ADP/ATP translocase SLC25A4 in more detail. Lastly, in contrast to a protein-centric enrichment approach, the analysis of different binding sites of both probes within single proteins, demonstrated here on EGFR and VDAC2, may aid in the identification of secondary binding sites or predict the binding poses of inhibitors. Insights from the PhosID-ABPP analysis of these two ABPs serve as a valuable resource for understanding drug on- and off-target engagement in a dose- and site-specific manner, elucidating the effect of DMAM addition on EGFR-directed inhibitors and contributing to the advancement of drug development efforts.

## Experimental Procedures

### Cell Culture

A431 and A549 cells (CRL-1555 and CCL-185, ATCC) with a passage number below 20 were cultured in growth medium [Dulbecco’s modified Eagle’s medium (Gibco) supplemented with 10% fetal bovine serum (HyClone GE) and 100 units/mL penicillin–streptomycin (Gibco)]. Cells were grown under a humidified atmosphere with 5% CO_2_ at 37 °C in T175 flasks (Greiner). Cells were split twice a week by washing with Dulbecco’s phosphate-buffered saline (DPBS, Lonza) and treated with 0.05% trypsin-EDTA (Gibco) for cell detachment. After detachment, trypsin was quenched by adding growth medium. 1/10 of the cell suspension was taken and grown with fresh growth medium in a new T175 flask.

### Activity-Based Probe Incubation in Cell Culture

Experiments were performed in triplicate (*n* = 3). 5 × 10^6^ cells were plated in 15 cm plates (Greiner) 1 day before probe incubation and maintained under a humidified atmosphere with 5% CO_2_ at 37 °C. The growth medium was replaced by treatment medium [growth medium with the corresponding concentrations (1 nM, 10 nM, 100 nM, 1.0 μM, 10 μM or 25 μM for individual probes) of PF-06672131 and/or PF-6422899 (Sigma-Aldrich)] and incubated at 37 °C, 5% CO_2_ for 4 h. Cells were washed with ice-cold DPBS, harvested using a cell scraper in 1 mL of ice-cold DPBS. Then, the cell suspension was spun down at 400 *g* for 5 min, and the supernatant was aspirated. The cell pellet was snap-frozen in liquid nitrogen and stored at −80 °C for later use.

### Cell Lysis

Cell pellets were lysed in 500 μL of lysis buffer per 15 cm plate, consisting of 50 mM HEPES (Sigma-Aldrich, pH 7.5), 0.5% NP-40 (Applichem), 0.2% SDS (Gen-Apex), 2 mM MgCl_2_ (Sigma-Aldrich), 10 mM NaCl (Merck), 1× protease inhibitor cocktail (Roche), and 0.5 μL/mL benzonase (Millipore). Cell lysates were incubated at RT for 15 min to allow DNA cleavage. Cell debris and DNA were spun down for 30 min at 20 567 *g* at 16 °C. The supernatant was collected, and the protein concentration was determined by a bicinchoninic acid assay (Thermo Fisher Scientific).

### Bioorthogonal Chemistry Reactions for Proteomics

The copper(I)-catalyzed azide–alkyne cycloaddition (CuAAC) was performed on 4 and 2.5 mg of protein lysates for timsTOF optimization and dual-probe analyses, respectively, in 2 M urea (Merck) in 1 × 50 mM HEPES (pH 7.5). CuAAC components were added in the following order: 5 mM tris(3-hydroxypropyltriazolylmethyl)amine (Lumiprobe), 2.5 mM CuSO_4_ 5·H_2_O (Sigma-Aldrich), 500 μM phosphonate-azide (prepared as described in van Bergen et al., 2023,^12^ and 25 mM sodium ascorbate (Sigma-Aldrich) in a final volume of 2 mL. Samples were incubated for 2 h at RT while rotating. Methanol–chloroform precipitation was performed to remove the CuAAC components, and the air-dried pellets were resuspended in 500 μL of 8 M urea and sonicated in a Bioruptor (Diagenode) with high amplitude for 10 min with cycles of 30 s on and 30 s off.

### Sample Processing for Digestion

Clicked and dissolved protein samples were diluted to 4 M urea with 50 mM ammonium bicarbonate (pH 8, AmBic, Sigma-Aldrich). The proteins were reduced with 4 mM DTT (Sigma-Aldrich) for 60 min at RT and alkylated in the dark using 8 mM iodoacetamide (Sigma-Aldrich) for 30 min. Residual iodoacetamide was quenched by adding DTT to a final concentration of 4 mM. Next, protease incubation (Pepsin Porcine, 1:50, enzyme to protein ratio, Sigma-Aldrich) was performed for 4 h at 37 °C in 40 mM HCl in a total volume of 2 mL (pH 2). Digested material was immediately desalted using 3 cc C18 Seppak cartridges (Waters) and air-dried using a vacuum centrifuge.

### Dephosphorylation

Samples were dephosphorylated prior to immobilized metal affinity chromatography (IMAC) enrichment. Desalted peptides were dissolved in 1 mL of 1 × CutSmart buffer (pH 8, New England BioLabs) and incubated with 50 units of alkaline phosphatase (calf intestinal, QuickCIP, New England BioLabs) overnight at 37 °C while shaking. After dephosphorylation, all peptides were again desalted using 3 cc C18 Seppak cartridges and air-dried using a vacuum centrifuge.

### Automated Fe^3+^-IMAC Enrichment

Probe-phosphonate-labeled peptides were enriched using 5 μL of Fe(III)-NTA (Agilent Technologies) in an automated fashion by the AssayMAP Bravo Platform (Agilent Technologies). Fe(III)-NTA (nitrilotriacetic acid) cartridges were primed at a flow rate of 100 μL/min with 250 μL of priming buffer [0.1% TFA, 99.9% acetonitrile (ACN)] and equilibrated at a flow rate of 50 μL/min with 250 μL of loading buffer (0.1% TFA, 80% ACN). The flow-through was collected into a separate plate. Dried peptides were dissolved in 200 μL of loading buffer and loaded at a flow rate of 2 μL/min onto the cartridge. Columns were washed with 250 μL of loading buffer at a flow rate of 20 μL/min, and the phosphonate-labeled peptides were eluted with 35 μL of ammonia (10%) at a flow rate of 5 μL/min directly into 35 μL of formic acid (10%). Flow-throughs and elutions were air-dried afterward and stored at −20 °C.

### LC–MS/MS

Prior to analysis, dried peptides were dissolved in 20 μL of 2% formic acid supplemented with 20 mM citric acid (Sigma-Aldrich). Subsequently, 4 and 45% of the IMAC-enriched peptides were injected for the MS method optimization and dual probe analysis, respectively. Peptides were separated by an Ultimate 3000 nanoUHPLC system (Thermo Fisher Scientific) equipped with an Aurora series column (75 μm × 25 cm, 1.6 μm, C18; Ion Opticks) heated to 50 °C by an external column oven (Sonation). The peptides were separated in the 72 min linear gradient (13.1 min at 3% B and 85.1 min at 30% B) at a flow rate of 400 nL/min using 0.1% FA in Milli-Q as solvent A and 0.1% FA in acetonitrile as solvent B. The LC system was coupled to a trapped ion mobility quadrupole time-of-flight mass spectrometer timsTOF HT (Bruker Daltonics) via a nanoelectrospray ion source CaptiveSpray (Bruker Daltonics).

Data acquisition on the timsTOF HT was performed using TIMSControl 4.0.5.0 and Compass HyStar 6.0.30.0 (Bruker Daltonics) starting from the DDA-PASEF method optimized for standard proteomics. This method utilized a capillary voltage of 1600 V, a nebulizer dry gas flow rate of 3.0 L/min at 180 °C, an MS/MS target intensity of 20 000 counts, and dynamic exclusion of precursor release after 0.4 min. Singly charged peptides were excluded by an active inclusion/exclusion polygon filter applied within the ion mobility over the *m*/*z* heatmap. Data were acquired in the range of 100–1700 *m*/*z* with 10 PASEF ramps (100 ms accumulation/ramp) with a total cycle time of 1.17 s. For timsTOF method optimization, the selected parameters were tested. Namely, the TIMS range of 0.6–1.6 and 0.7–1.3 Vs/cm^2^, precursor intensity threshold of 1500 and 2500, combinations of linearly interpolated ion mobility-dependent collision energies (20, 30 eV at 0.6 Vs/cm^2^, and/or 60, 80 eV at 1.6 Vs/cm^2^), and precursor charge restriction 2+ to 5+ or 3+ to 5+ (methods A–G are summarized in Figure 1B). For subsequent concentration-dependent dual-probe experiments, method D was used (TIMS range of 0.7–1.3 Vs/cm^2^, precursor intensity threshold of 1500, CE of 30–60 eV, and charge states of 2+ to 5+).

### Database Search and Analysis

LC–MS/MS run files were searched against the human (20 375 entries) SwissProt database (version September 2020) using Fragpipe v19.1 with MSFragger 3.7, IonQuant 1.8.10, and Philosopher 4.8.1 search engines using default settings.^52^ The integrated Fragpipe contaminant database was used for filtering out contaminants. The cleavage site was set to nonspecific, and a peptide length between 5 and 30 was allowed. Oxidation of methionine, acetylation of the protein N-terminus, and carbamidomethylation of cysteines were set as variable modifications. PF-06672131-phosphonate (689.20422 Da) and PF-6422899-phosphonate (632.14632 Da) adducts were also set as variables in modification on cysteine. All modifications were used in the first search. Precursor and fragment mass tolerance were set to 20 and 50 ppm, respectively. The false discovery rate for PSMs and proteins was set to 1% using a target-decoy approach.

### Data Analysis, Statistical Analysis, and Visualization

In the optimization for the timsTOF HT and the analysis of the characteristics of both probes, the “psm.tsv” tables were used for analysis, and all psm.tsv data were combined in a table with R. The data were graphed in GraphPad Prism 9.5.1. The quantitative dual-probe analysis was performed in Skyline-daily 22.2.1.542. The “.pep.xml” and “.d” files were loaded in Skyline-daily to enable MS1 quantification and MS/MS visualization. An ion mobility library was generated based on the results in the 10 μM probe concentration in A431 and A549 cells, and the corresponding peaks were integrated at similar retention times in lower probe concentrations. Precursor peaks were filtered based on the isotope dot product (idotp) score of 0.9 in Skyline-daily. The filtered results were exported as a result table and further processed in RStudio 2023.6.2.561.^53^ Peptides without detection by a PSM in two out of three replicates at the 10 μM probe concentration were filtered out. Then, precursor peaks were filtered for presence in 2 out of the 3 replicates per condition. Afterward, the data were filtered for continuity over the concentrations on the modified peptide level (i.e., if a modified peptide was not found in a concentration, all values in the concentration below were filtered out). Then, to calculate the intensity per binding site, the summed MS1 peak areas of all peptides derived from the ABP binding sites were taken. Further processing and analysis of data were done in RStudio and Excel 2016 and visualization of graphs was done in GraphPad Prism 9. All spectra were exported from Skyline-daily and 3D protein modeling was conducted using UCSF ChimeraX 1.6.1.^54^ The figures were compiled and visualized using Adobe Illustrator 27.0.

### Cloning, Isolation, and Reconstitution of Murine ANT1

The cloning, isolation, and reconstitution of murine ANT1 followed an established protocol.^47^ In brief, for protein expression, we used the *E. coli* strain Rosetta (DE3; Novagen), and the protein was expressed in inclusion bodies, which were isolated by centrifugation of bacterial cells disrupted by the high-pressure One Shot Cell Disruptor (Constant Systems Limited, Daventry, UK) at 1 kbar. For reconstitution, the protein from the inclusion bodies was first solubilized in 100 mM Tris at pH 7.5, 5 mM EDTA, 10% glycerin (TE/G-buffer) containing 2% sodium lauryl sulfate, and 1 mM DTT. It was then gradually mixed with the membrane-forming lipids (DOPC, DOPE and CL; 45:45:10 mol %) dissolved in TE/G-buffer containing 1.3% Triton X-114, 0.3% *n*-octylpolyoxyethylene, 1 mM DTT, and 2 mM GTP. After several dialysis steps, the mixture was dialyzed three times against assay buffer (50 mM Na_2_SO_4_, 10 mM MES, 10 mM Tris, and 0.6 mM EGTA at pH 7.35). The dialyzate was centrifuged and passed through a hydroxyapatite column (Bio-Rad, Munich, Germany) to remove aggregates. Nonionic detergents were removed using Bio-Beads SM-2 (Bio-Rad).

The protein concentration of the proteoliposomes was measured with the Micro BCA protein assay kit (Thermo Fisher Scientific, Vienna, Austria). SDS-PAGE and silver staining verified protein purity (Figure S7). The following batches of proteins were used in this study: #55 and #56.

### Substrate Exchange Rate Measurements of ANT1

ANT1-containing proteoliposomes were filled with 2 mM ATP (dissolved in the assay buffer at pH 7.34) and 2 mM ^3^H-labeled ATP prior to extrusion. Unilamellar liposomes were formed using a small-volume extruder (Avanti Polar Lipids, Alabaster, Alabama, USA) with 100 nm pore filters (AVESTIN, Europe, Mannheim, Germany). 1 mM of DTT (1 mM) was added to proteoliposomes after extrusion to prevent any free sulfhydryl group-mediated aggregation. ANT1-facilitated transport was initiated by adding 2 mM ADP (dissolved in the assay buffer at pH 7.34) and stopped immediately at the corresponding times by the addition of carboxyatractyloside (CATR) and bongkrekic acid (BKA), 10 nM each (dissolved in the assay buffer at pH 7.34). The samples were then subjected to size exclusion chromatography on Sephadex^TM^ G-50 dextran gels. Remaining radioactivity in proteoliposomes was measured by liquid scintillation counting (Tri-Carb 2100TR, PerkinElmer, Waltham, MA, USA). In the case of inhibition, 10 μM Afatinib or Afatinib-alkyne (PF-06672131) were added to proteoliposomes prior to extrusion to account for the random orientation of ANT1 in the membrane. Afatinib and Afatinib-alkyne were dissolved in DMSO. For control measurements, the same protocol was used with empty liposomes. The ADP/ATP exchange rates were calculated as described previously.^55^

For all measurements, membranes were prepared with DOPC:DOPE:CL (45:45:10 mol %). Lipid and protein concentrations were 1.5 mg mL^–1^ and 4 μg/(mg of lipid), respectively. The assay buffer solution consisted of 50 mM Na_2_SO_4_, 10 mM Tris, 10 mM MES, and 0.6 mM EGTA at pH = 7.34 and *T* = 32 °C.

## Supporting Information Available

The mass spectrometry proteomics data have been deposited to the ProteomeXchange Consortium via the PRIDE^56^ partner repository with the data set identifier PXD045864
Supplementary Figures S1−S8 (PDF)
Information on peptide identifications in optimization LC–MS runs on timsTOF HT, supporting data 1 (XLSX)
Information on peptide identifications and quantifications of PF131- and PF899-bound peptides in the dual-probe PhosID-ABPP analysis, supporting data 2 (XLSX)

## Nomenclature

ABP: Activity-based probe
ABPP: Activity-based protein profiling
CID: Collision-induced dissociation
CTSC: Cathepsin C
CuAAC: Copper(I)-catalyzed azide–alkyne cycloaddition
DMAM: Dimethylaminomethyl
DPBS: Dulbecco’s phosphate-buffered saline
DUS2: tRNA-dihydrouridine synthase
EGFR: Epidermal growth factor receptor
GMPS: Guanosine monophosphate synthase
idotp: Isotope dot product
IMAC: Immobilized metal affinity chromatography
LC–MS: Liquid chromatography—mass spectrometry
NTA: Nitrilotriacetic acid
PF131: PF-06672131
PF899: PF-6422899
PSM: Peptide-spectrum match
SLC25A4/5/6: Solute carrier 25A4/5/6
SOAT1: Sterol O-acyltransferase 1
TIMS: Trapped ion mobility spectrometry
VDAC2: Voltage-dependent anion channel 2
