# Supplementary material for: Dual-Probe Activity-Based Protein Profiling Reveals Site-Specific Differences in Protein Binding of EGFR-Directed Drugs
Source: ACS Chem Biol. 2024 Jul 25;19(8):1705–18. doi: 10.1021/acschembio.3c00637 (PMC11334109; doi:10.1021/acschembio.3c00637)
Supplement: Supplementary file 1 — cb3c00637_si_001.pdf [file cb3c00637_si_001.pdf]

Supporting information

Dual-Probe Activity-Based Protein Profiling Reveals Site-Specific Differences in Protein Binding of EGFR-Directed Drugs

Wouter van Bergen[1,2], Kristina Žuna[3], Jan Fiala[1,2], Elena E. Pohl[3], Albert J.R. Heck [1,2], Marc P. Baggelaar[1,2*]

[1] Biomolecular Mass Spectrometry and Proteomics, Bijvoet Center for Biomolecular Research and Utrecht Institute for Pharmaceutical Sciences, University of Utrecht, Padualaan 8, Utrecht 3584 CH, The Netherlands

[2] Netherlands Proteomics Center, Padualaan 8, Utrecht 3584 CH, The Netherlands

[3] Physiology and Biophysics, Department of Biological Sciences and Pathobiology, University of Veterinary Medicine, Vienna, Austria [*] Contact details for correspondence: m.p.baggelaar@uu.nl

Supporting Figures and Data

Figure S1. TimsTOF analysis enables efficient and specific detection of activity-based PF131 binding sites.

Figure S2. Comparative dose-dependent profiling of multiple ABPs in a single complex proteome reveals probe-specific characteristics.

Figure S3. Dose-dependent site-specific target landscape reveals probe-specific target engagement in intact A549 cells.

Figure S4. Concentration-dependent quantitative analysis of ABP binding to EGFR;C797 and off-target sites.

Figure S5. Dose-dependent profiling of ABP binding sites.

Figure S6. Quantitative analysis of ABP binding sites on VDAC1 and VDAC3.

Figure S7. Graphical overview of the ATP transport assay

Figure S8. ANT1 expression, purification and ATP transport inhibition by known inhibitors

Appendix 1. Calculation of the transport rates.

Supplemental Data 1. Identifications in optimization LC-MS runs on timsTOF HT. (XLSX)

Supplemental Data 2. Identifications and quantifications of PF131- and PF899-bound peptides in the dual-probe PhosID-ABPP analysis. (XLSX)

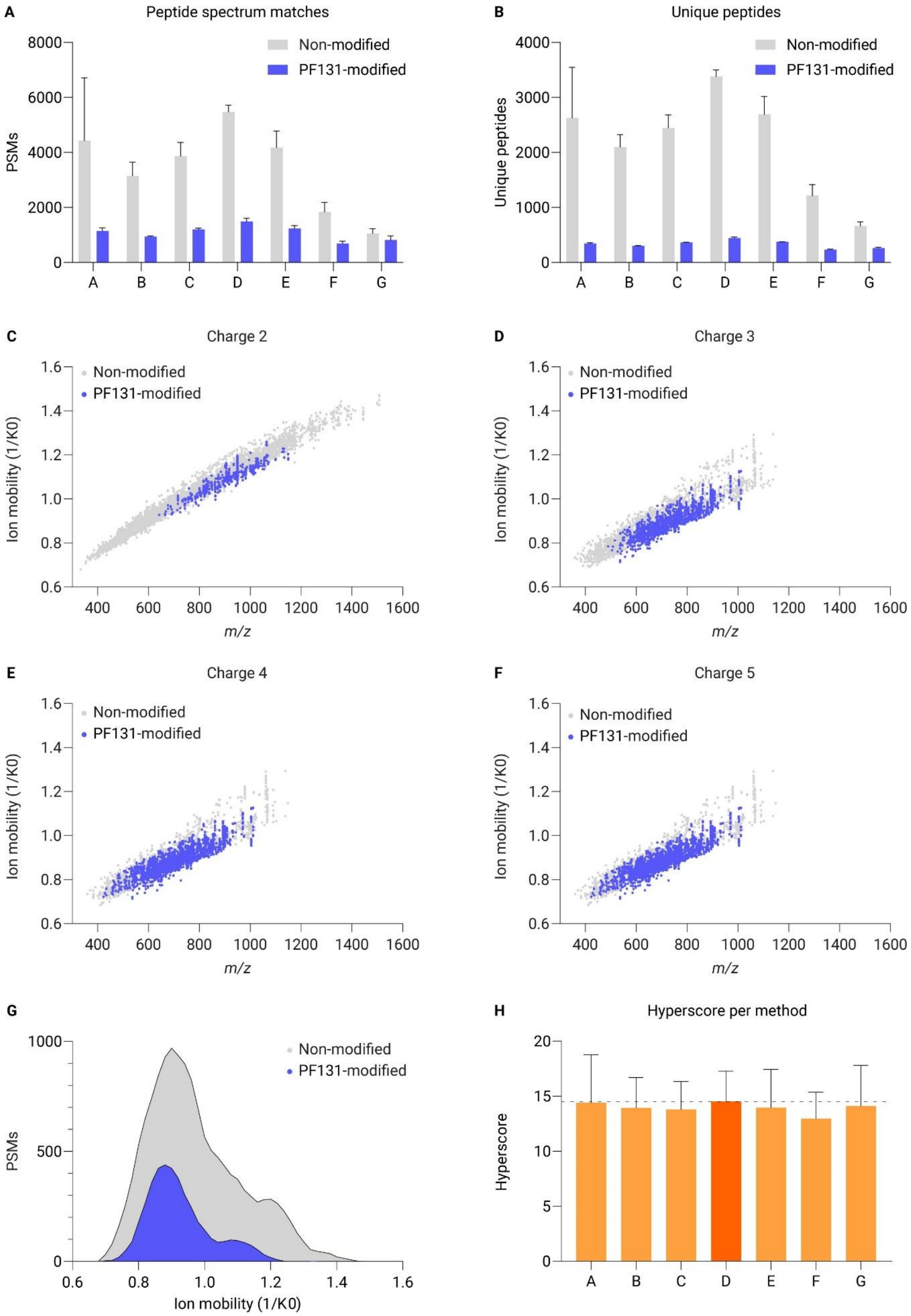
A
Peptide spectrum matches
PSMs
Non-modified
PF131-modified
B
Unique peptides
Unique peptides
C
Charge 2
D
Charge 3
E
Charge 4
F
Charge 5
Ion mobility (1/K0)
m/z
G
PSMs
Ion mobility (1/K0)
H
Hyperscore per method
Hyperscore

S3

Figure S1: TimsTOF analysis enables efficient and specific detection of PF131 binding sites. **A)** The peptide-spectrum matches (PSMs) of non-modified and PF131-modified peptides using the different methods described in Figure 1A. **B)** The number of unique non-modified and PF131-modified peptides using the different methods described in Figure 1A **C-F)** Plots displaying the ion mobility versus m/z of non-modified peptides and PF131-modified peptides detected through method A for different charge states (charge 2+ to 5+). **G)** The PSM distribution of the ion mobility for non-modified (gray) and PF131-modified (blue). **H)** Bar graph displaying the average of the hyperscores for PF131-modified peptides using the different methods described in Figure 1A. Method D shows the highest average hyperscore with a score of 14.5. Hyperscore is a measure of peptide identification quality in MS-fragger.(doi: 10.1038/nmeth.4256)

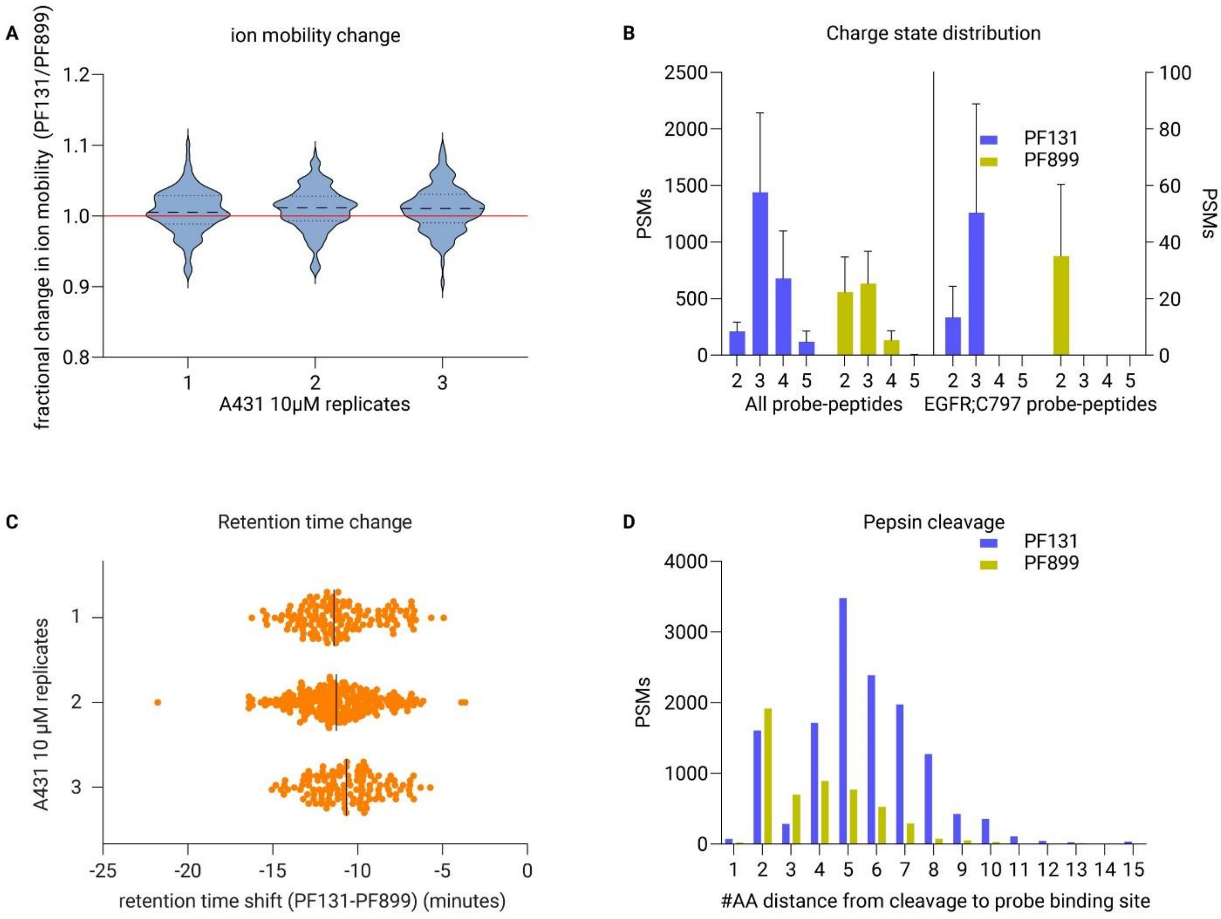

Figure S2: Comparative dose-dependent profiling of multiple ABPs in a single complex proteome reveals probe-specific characteristics. **A)** Fractional change in ion mobility PF131- and PF899-labeled peptides with the same sequence in the 10 µM ABP concentration in A431 cells. The red line indicates no change in ion mobility. **B)** Peptide-spectrum matches (PSMs) corresponding to different charge states of peptides labeled with PF131 (blue) and PF899 (yellow). The left section displays the charge distribution over the entire population of ABP-labeled peptides, while the right section shows the peptides spanning the EGFR;C797 site. **C)** Shift in retention time between PF131- and

PF899-labeled peptides of the same sequence in the 10 µM probe concentration in A431 cells. **D)** Distribution of peptide-spectrum matches (PSMs) corresponding to amino acid distance from the pepsin cleavage site to the ABP-bound cysteine for peptides bound to PF131 (blue) and PF899 (yellow).

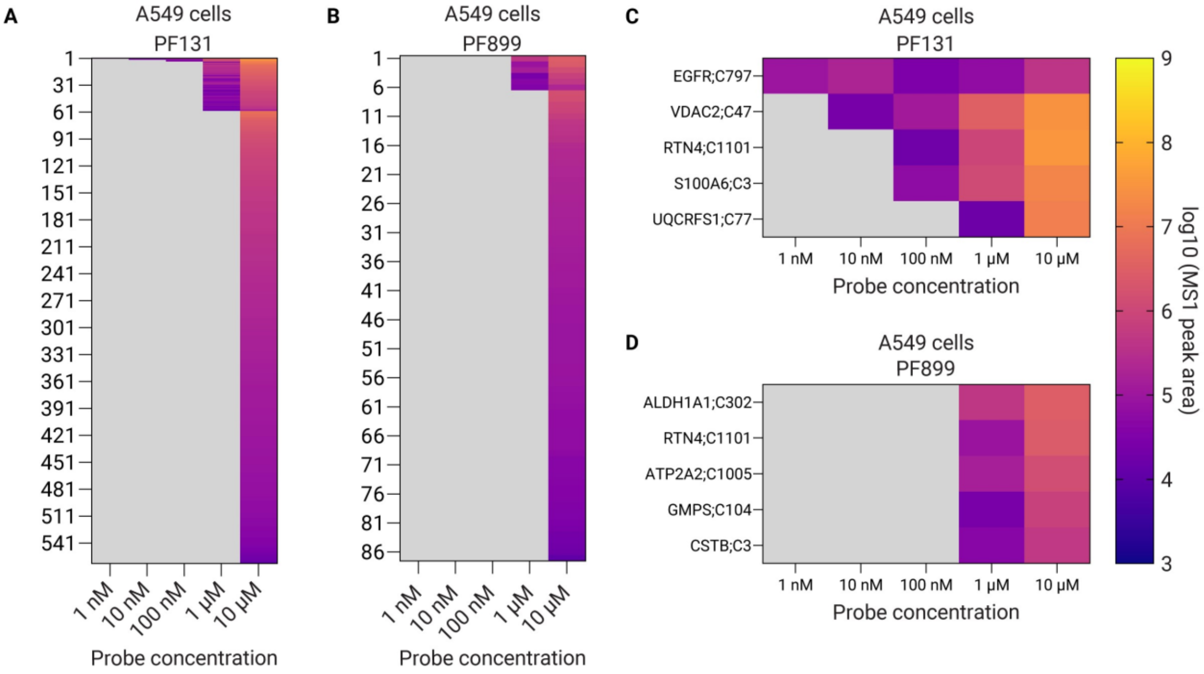

Figure S3: Dose-dependent site-specific target landscape reveals probe-specific target engagement in intact A549 cells. **A)** Heatmap displaying the average aggregated MS1 peak areas per PF131 binding site at different concentrations in A549 cells. **B)** Heatmap displaying the average aggregated MS1 peak areas per PF899 binding site at different concentrations in A549 cells. **C)** A zoom of the top-5 PF131 binding sites from A. **D)** A zoom of the top-5 PF899 binding sites from B.

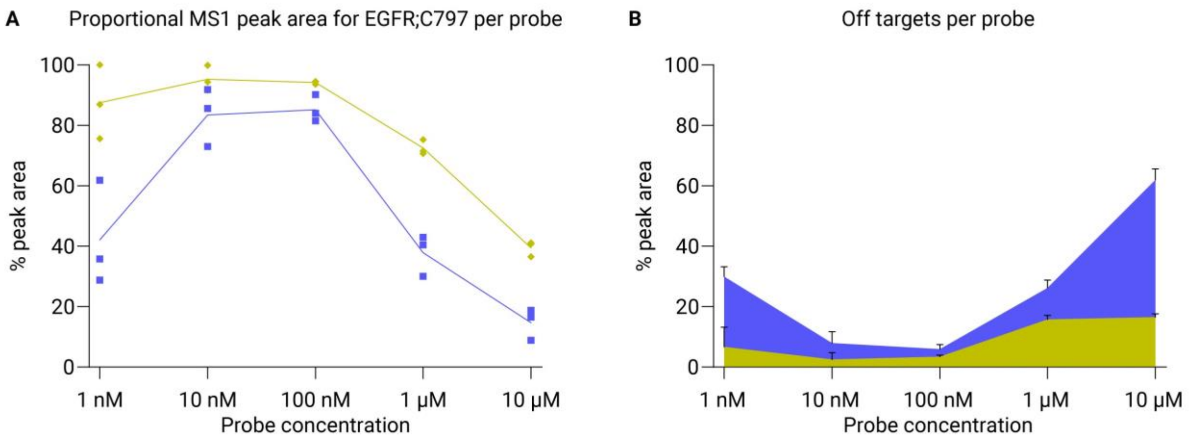

Figure S4: Concentration-dependent quantitative analysis of ABP binding to EGFR;C797 and off-target sites in A431 cells. **A)** Line plot of the proportional aggregated MS1 peak areas per probe (i.e., the intensity of the specific site as a percentage of the specific probe-labeled intensity in the LC-MS experiment) for PF131 (blue) and PF899 (yellow) binding to EGFR;C797 across the concentration range. **B)** Density plot of the sum of aggregated MS1 peak areas for PF131 (blue) and PF899 (yellow) binding to any site other than EGFR;C797 across the concentration range, corresponding to the gray density in Figure 3D.

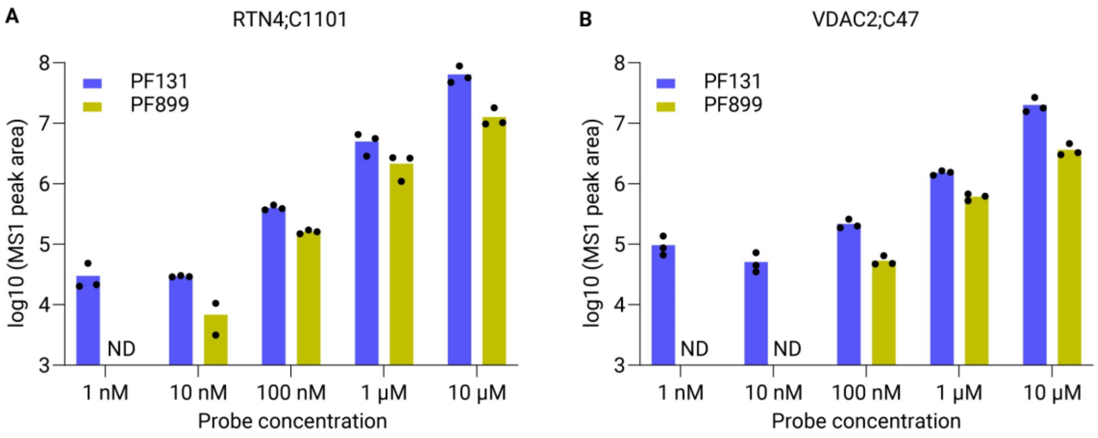

Figure S5: Dose-dependent profiling of ABP binding sites in A431 cells. **A/B)** Bar graph of the aggregated MS1 peak areas for PF131 (blue) and PF899 (yellow) binding to RTN4;C1101, and VDAC2;C47 across the concentration range. ND; not detected.

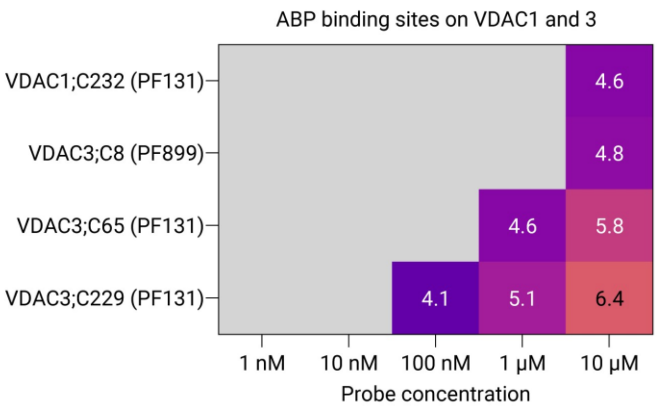

Figure S6: Quantitative analysis of ABP binding sites on VDAC1 and VDAC3 in A431 cells. Heatmap displaying the log10 average aggregated MS1 peak areas per ABP binding site on VDAC1 and 3 at different concentrations in A431 cells.

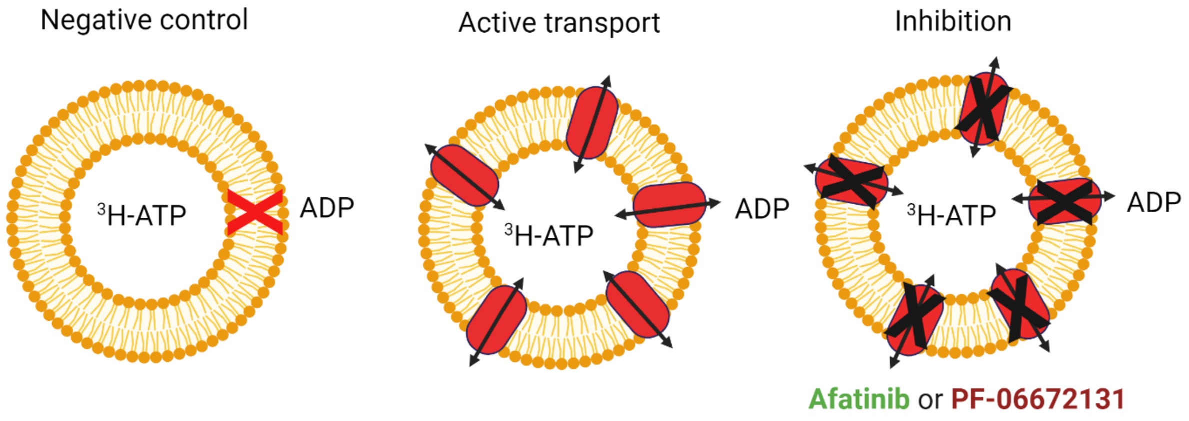

Figure S7: Graphical overview of the ANT1 mediated ATP transport assay.

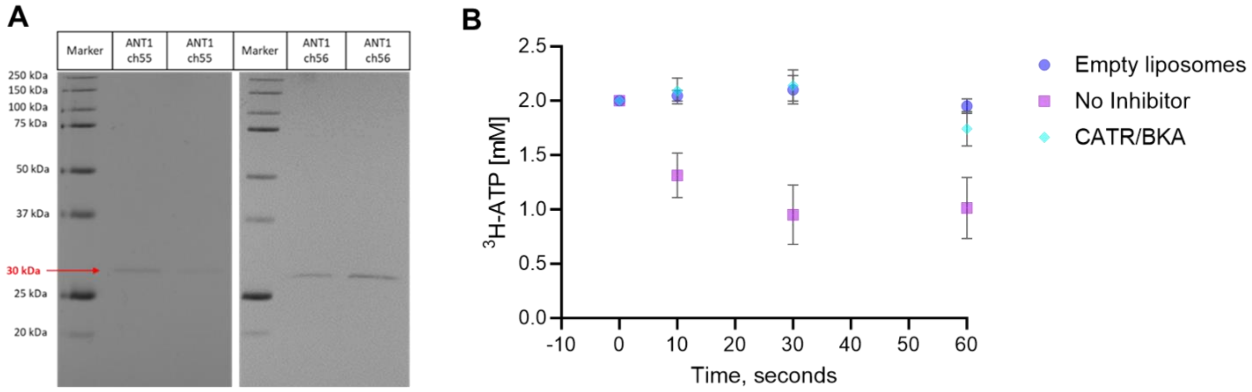

figure S8: A. Representative silver staining of ANT1 reconstituted into proteoliposomes. Proteoliposomes were loaded in separate lanes on a 15% acrylamide gel, separated by SDS-PAGE and visualized by silver staining. Reconstituted ANT1 appears at the expected size (~30 kDa, red arrow). Precision Plus Protein Dual Color Standard was loaded as a molecular weight marker. **B**. Decrease of ^{3}H-ATP concentration upon initiation of the ATP/ADP exchange in proteoliposomes containing reconstituted SLC25A4 (squares). Transport was not present in empty liposomes (circles) and active transport was observed in proteoliposomes containing reconstituted SLC25A4 (ANT1). Transport was inhibited by known SLC25A4 inhibitors as combined carboxyatractyloside (CATR) and bongkrekic acid (BKA) incubation, each at 10 nM (red triangles) blocked ^{3}H-ATP transport.

Appendix 1: Calculation of the ATP transport rates

After measurement of the counts per minute (CPM) in different elution fractions with the liquid scintillation counter, we calculated ΔCPM by subtracting the background signal measured in elution fractions 1 and 2 without liposomes (CPM_{min}) from the signal obtained in elution fractions containing (proteo-)liposomes (CPM_{max})(1). Calculated transport rates in µMol of 3H-ATP/(s*(mg protein): No inhibitor (0.72 ± 0.28), PF131 (0.06 ± 0.01), Afatinib (0.42 ± 0.02).

$$\Delta CPM = CPM_{max} - CPM_{min} \quad (1)$$

The remaining concentration of radioactive substrate inside the (proteo-) liposomes for each time condition (t = 0, 10 s, 30 s, 60 s) was determined by (2). where $[P_i](t)$ denotes the concentration of intraliposomal P_i after time t. $[P_i](t = 0)$ is the initial concentration of 2 mM, CPM(t) is the CPM value measured in the sample at time t, and CPM (t = 0) is the CPM value measured at t = 0 s.

$$[P_i](t) = P_i(t=0)\frac{CPM(t)}{CPM(t=0)} \quad (2)$$

The resulting data were plotted using Sigma Plot 12.5 (Systat Software GmbH, Erkrath, Germany) and presented as the mean ± SD of at least three independent measurements. The transport rate was determined by exponential fitting of the curve and plotted accordingly. The fit was performed according to (3), where $[P_i](t)$ is the concentration of intraliposomal P_i at time t, $[P_i](t = 0)$ is the initial, predetermined concentration of 2 mM, and a and b are the coefficients determined by the performed fit.

$$[P_i](t) = P_i(t=0) + ae^{-b\cdot t} \quad (3)$$

Subsequently, the function of the exponential fit was differentiated to determine the initial transport rate and the associated standard deviation of a substrate k_s, as shown in (4), (5) and (6), where k_s is the initial transport rate at t=0, a and b are coefficients determined by the performed fit, and Δa and Δb are the associated standard deviations.

$$k_s = \frac{\partial[Pi](t)}{\partial t} at\ (t=0) \quad (4)$$

$$k_s = -a\cdot b\cdot e^{-bt}, thus\ k_s = a\cdot b\ at\ t = 0 \quad (5)$$

$$\Delta k_s = \sqrt{\frac{(\Delta a)^2}{a^2} + \frac{(\Delta b)^2}{b^2}} \quad (6)$$

The calculated transport rate and the associated standard deviation was then adjusted to the protein concentration in the sample in (7) and (8). where $k_{SLC25A4}$ refers to the transport rate in mmol per min and mg SLC25A4, and ρ SLC25A4 being the mass of SLC25A4 inside the sample.

$$\Delta k_{\mathrm{SLC25A4}} = \frac{k_s}{\rho_{SLC25A4}} \quad (7)$$

$$\Delta k_{\mathrm{SLC25A4}} = \sqrt{\frac{(\Delta k_s)^2}{k_s^2} + \frac{(\Delta\rho_{\mathrm{SLC25A4}})^2}{\rho_{\mathrm{SLC25A4}}^2}} \quad (8)$$
